# Synthesis, characterization, *in vitro* and computational assessment of new xanthene hydrazone derivatives as promising vasorelaxant agents

**DOI:** 10.1039/d6ra01067a

**Published:** 2026-04-17

**Authors:** Mohammed El Mesky, Ismail bouadid, Fatimazahra Guerguer, Hicham Zgueni, Yassine Rhazi, Mohammed Chalkha, Farid Khallouki, Samir Chtita, Sahar Abdulaziz AlSedairy, Mourad A. M. Aboul-Soud, John P. Giesy, Driss Chebabe, El Houssine Mabrouk, Mohamed Eddouks

**Affiliations:** a Laboratory of Materials Engineering for the Environment and Natural Resources, Faculty of Sciences and Techniques, Moulay Ismail University of Meknès B.P. 509, Boutalamine 52000 Errachidia Morocco m.elmesky@edu.umi.ac.ma mohammed.chalkha1@usmba.ac.ma; b Team of Ethnopharmacology and Pharmacognosy, Faculty of Sciences and Techniques Errachidia, Moulay Ismail University of Meknes Errachidia Morocco; c Laboratory of Analytical and Molecular Chemistry, Faculty of Sciences Ben M'Sik, Hassan II University of Casablanca Casablanca Morocco; d Laboratory of Engineering of Organometallic, Molecular Materials, Environment, and Innovative Pedagogy (LIMOMEPI), Faculty of Sciences Dhar EL Mahraz, Sidi Mohamed Ben Abdellah University P.O. Box 1796 (Atlas) 30000 Fez Morocco; e Department of Food Sciences and Nutrition, College of Food and Agricultural Sciences, King Saud University P.O. Box 2460 Riyadh 11451 Saudi Arabia; f Center of Excellence in Biotechnology Research (CEBR), College of Applied Medical Sciences, King Saud University P.O. Box 17 10219 Riyadh 11433 Saudi Arabia maboulsoud@ksu.edu.sa; g Department of Veterinary Biomedical Sciences and Toxicology Centre, Western College of Veterinary Medicine, University of Saskatchewan Saskatoon SK S7N 5B4 Canada Jgiesy@aol.com; h Department of Integrative Biology and Center for Integrative Toxicology, Michigan State University East Lansing MI 48824 USA; i Department of Environmental Sciences, Baylor University Waco 76706 USA

## Abstract

Functionalized xanthene hydrazone derivatives were synthesized using two distinct pathways. Two routes of synthesis, involving condensation and *O*-alkylation reactions, were adopted to synthesize the target compounds. The structures of the synthetised intermediates and xanthene hydrazone derivatives were confirmed through high-resolution mass spectrometry (HRMS), ^1^H-nuclear magnetic resonance (NMR), and ^13^C-NMR. Vasorelaxation assays indicated that derivatives modified solely by alkylation or condensation remained inactive. It was observed that compounds became active only when both functional groups were present simultaneously, highlighting the importance of the complementarity of the ester and hydrazone in the mechanism of action. Among the derivatives examined, compounds F3 and F4 exhibited the greatest potency as vasorelaxants, with EC_50_ values of 38.204 and 41.300 µg mL^−1^ and *E*_max_ values of 80.61% and 83.13%, respectively. While F3 and F4 displayed somewhat lower potency than verapamil (EC_50_ = 18.000 ± 1.00 µg mL^−1^; *E*_max_ = 61.67 ± 4.10%), they demonstrated significantly greater maximal effectiveness. These findings highlighted the great potential of compounds F3 and F4 as new vasorelaxant agents. Results of the *in vitro* tests on rat aortic rings that were pre-contracted using epinephrine revealed a clear correlation between both the chemical structure and biological activity. The results of molecular docking and molecular dynamics simulations show that F3 and F4 formed stable and robust binding modes with the human L-type calcium channel (CaV1.2). Furthermore, the *in silico* findings suggest that F3 and F4 possess acceptable ADMET properties, supporting their potential as promising drug candidates. Overall, the results indicate that these functionalized xanthene-hydrazone derivatives represent a promising foundation for the development of novel vasorelaxant agents.

## Abstract 2

This article reports the synthesis of novel xanthene-hydrazone derivatives, their structural characterization, and the evaluation of their vasorelaxant properties using both *in vitro* and *in silico* approaches.

## Graphical abstract

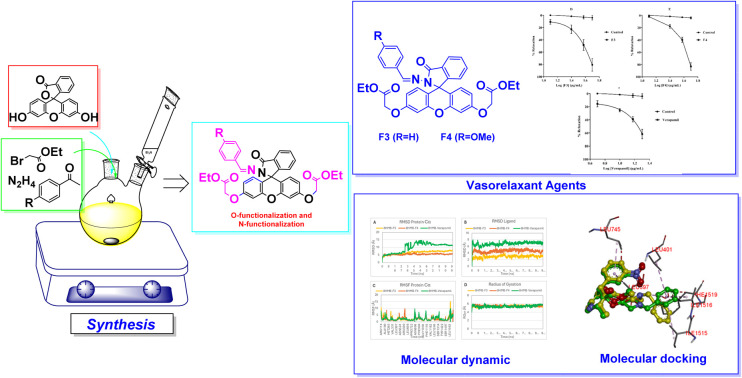

## 1. Introduction

Blood pressure is essential for adequate perfusion of vital organs. It depends on two main mechanisms: the resistance of blood vessels,^1^ which depends on the degree of vascular contraction, and cardiac output, which is linked to the frequency and strength of the heartbeat. When blood pressure falls to abnormally low levels, tissues lack oxygen, which can impair organ function. Conversely, chronic hypertension eventually damages the walls of blood vessels and gradually affects the heart, brain, kidneys and even the eyes.

Hypertension is defined as persistently elevated blood pressure, according to the WHO. It is a major health problem that affects more than one billion individuals worldwide and leads to nearly 10 million deaths each year.^2^ It is frequently referred to as the ‘silent killer’ since it usually does not show any warning signs for years. If left undetected and untreated, it can result in severe complications such as heart problems, strokes, kidney disease and retinal damage.^3^

Antihypertensive drugs are the standard treatment for high blood pressure. They are effective in controlling blood pressure and reducing cardiovascular risks. However, long-term use is often associated with side effects such as palpitations, persistent dry cough, constant fatigue, and even kidney complications. For many patients, these side effects make long-term adherence to treatment difficult.

One of the key mechanisms for combating hypertension is the relaxation of the smooth muscles of the blood vessels. This is why research is focusing on the development of more potent and better-tolerated vasorelaxant agents.^4–6^ The design of hybrid molecules, which combine several pharmacophores in a single structure, appears particularly promising. The concept is to combine complementary mechanisms capable of acting simultaneously on several biological pathways. By carefully assembling these elements, it is possible to improve the potency, selectivity and tolerance of the treatment, paving the way for a new generation of antihypertensive drugs that are both more effective and safer.^7,8^

**Fig. 1:**
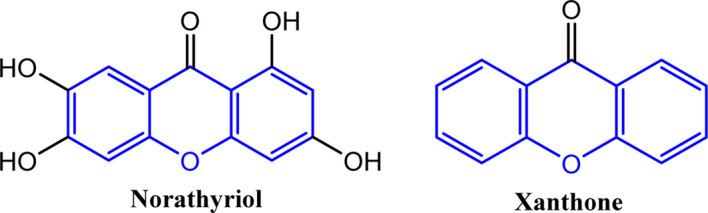
Xanthene derivatives exhibiting vasorelaxant activity.

In medicinal chemistry, the xanthene scaffold is considered highly versatile. For years, researchers have been exploring its derivatives, xanthones, fluorescein, rhodamine, with continuous discovery of new properties. These molecules exhibit a wide range of biological activities: antiviral,^9^ antihyperlipidemic effects,^10^ antibacterial action,^11^ anti-inflammatory,^12^ antimalarial activity,^13^ antimicrobial,^14^ anticancer,^15^ antileukemic,^16^ free radical scavenging,^17^ antitumor,^18^ and even capable of fighting certain cancers or promoting apoptosis. Some, such as certain xanthones and norathyriol, also show interesting potential for regulating blood pressure (Fig. 1).^19^

**Fig. 2:**
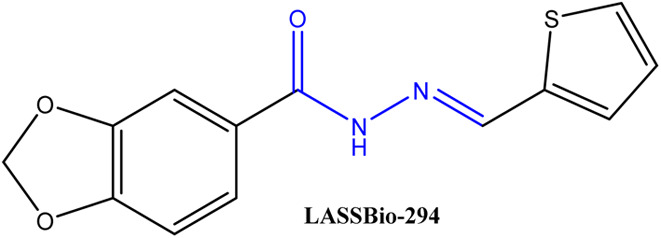
LASSBio-294 exhibiting vasorelaxant activity.

Recently, hydrazones and their derivatives have attracted considerable interest. They are relatively simple to synthesize and have a wide range of pharmacological effects.^20^ They possess a flexible and stable structure capable of forming hydrogen bonds. This explains their broad pharmacological activities: antihypertensive,^21^ antibacterial,^22^ antifungal,^23^ antiviral and even anticancer.^24,25^ By combining them with other functional groups, hybrid molecules with unique physicochemical properties can be obtained (Fig. 2). Their versatility often allows them to target several mechanisms at once, improving both their efficacy and selectivity. For these reasons, they are considered promising candidates for the design of new multifunctional drugs.^26,27^

Xanthene derivatives have interesting properties, particularly due to their ester and hydrazone groups. Therefore, we designed and synthesized new functionalised xanthene-based molecules and evaluated their vasorelaxant activity. Two synthesis routes were successfully used, and all compounds, both intermediates and final products, were characterised by NMR spectroscopy and high-resolution mass spectrometry. To investigate their vasorelaxant effects, we tested the compounds step by step, first the intermediates, then the final hybrids. This stepwise approach allowed us to better understand how the order of functionalisation influences the pharmacological behaviour of xanthene-hydrazone compounds. The results obtained provide valuable insights for improving and selecting the most promising candidates as potential vasodilators. In addition to the experiments, we conducted computational studies, including molecular docking and molecular dynamics simulations, to better understand the interactions with biological targets. An ADMET analysis was also performed to assess the therapeutic potential of these molecules.

## 2. Materials and methods

### 2.1. Chemistry

Melting points of compounds were determined by use of a Kofler bank. The evolution of the synthesis was determined by thin-layer chromatography (TLC) on silica 60 F254 (E. Merck). NMR spectra were measured on a Bruker Advanced 300 WB at 300 MHz and 600 MHz for solutions in DMSO-d6, with chemical shifts reported in *δ* ppm relative to tetramethyl silane 7 (TMS) as an internal control. Mass spectra were obtained by using an UltiMate 3000 (Thermo Scientific).

### 2.2. Synthesis of FH and F1–7

#### 2.2.1. Synthesis of 2-amino-3′,6′-dihydroxyspiro[isoindolin-3-one-1,9′-xanthene] FH

5 mmol (1.742 g) of fluorescein lactone was dissolved in 30 mL of ethanol in a 100 mL round-bottom flask, then stirred for 30 minutes at 80 °C. The reaction was then carried out under magnetic stirring during reflux after 15 mmol of hydrazine hydrate was introduced. TLC was applied to track the reaction's progress. After the reaction was complete, the mixture was diluted with 100 mL of cold water, the precipitate was collected by filtration, dried, and then purified by column chromatography using a 2 : 1 ethyl acetate/hexane combination. Product FH was obtained as a fragile, white solid.

The yield was 76% of a light off-white solid; ^1^H NMR (500 MHz, DMSO-d6): *δ* (ppm) = 4.40 (s, 2H, NH_2_), 9.02 (s, 2H, 2(–OH)), 7.91–7.85 (m, 1H, ArH), 7.58 (dtd, *J* = 21.4, 7.4, 1.2 Hz, 2H, ArH), 7.38 (dd, *J* = 6.8, 3.0 Hz, 2H, ArH), 7.31 (dd, *J* = 5.1, 1.9 Hz, 3H, ArH), 7.10 (dd, *J* = 7.4, 1.2 Hz, 1H, ArH), 6.62 (d, *J* = 2.3 Hz, 2H, ArH), 6.46 (d, *J* = 8.6 Hz, 2H, ArH), 6.42 (dd, *J* = 8.6, 2.4 Hz, 2H, ArH); ^13^C NMR (126 MHz, DMSO-d6): *δ* (ppm) = 164.17 (C=O), 159.13, 152.81, 150.91, 149.80, 135.04, 134.52, 130.89, 129.66, 129.65, 129.36, 128.54, 127.29, 124.36, 123.73, 112.85, 110.75, 103.01 (aromatic carbons), 65.93 (spiro carbon); HRMS (*m*/*z*): calculated for C_20_H_14_N_2_O_4_ 345.09356, found [M–H]^−^: 345.08569.

#### 2.2.2. Synthesis of diethyl 2,2′-((2-aminospiro[isoindolin-3-one-1,9′-xanthene]-3′,6′-diyl)bis(oxy))diacetate F1

To obtain compound F1, 1 mmol of fluorescein hydrazine FH compound was dissolved in 20 mL of DMF in a 100 mL round-bottom flask. Next, 2.2 mmol of K_2_CO_3_ and 0.1 mmol of BTBA were added. At room temperature, the resulting mixture was stirred for 30 min, followed by the addition of 2.2 mmol of ethyl bromoacetate. The reaction was allowed to continue at ambient temperature while being stirred magnetically. The progress of the reaction was monitored by use of TLC. After the reaction was complete, 100 millilitres of cold water were used to dilute the mixture. Filtration was used to separate the precipitate, which was subsequently dried and purified using column chromatography.

The yield was 87%, a yellow solid, m. p.: 168–170 °C, f. r. = 0.52; ^1^H NMR (500 MHz, DMSO-d6): *δ* 7.77 (dd, *J* = 5.7, 3.1 Hz, 1H), 7.47 (dd, *J* = 5.7, 3.0 Hz, 2H), 7.00–6.94 (m, 1H), 6.78 (d, *J* = 2.6 Hz, 2H), 6.61 (dd, *J* = 8.5, 2.6 Hz, 2H), 6.51 (d, *J* = 8.8 Hz, 2H), 4.78 (s, 4H), 4.43 (s, 2H), 4.14 (q, *J* = 7.1 Hz, 4H), 1.21–1.15 (m, 6H); ^13^C NMR (126 MHz, DMSO-d6): *δ* 169.03, 166.17, 158.87, 152.79, 151.67, 133.32, 129.78, 129.24, 128.65, 123.97, 123.07, 112.86, 112.18, 102.32, 65.43, 64.95, 61.26, 14.57.

#### 2.2.3. Synthesis of compounds F5–F7

The syntheses of compounds F5, F6 and F7 started by dissolving 1 mmol of fluorescein hydrazine FH in 20 mL of ethanol in a 100 mL round-bottom flask. After adding a few drops of glacial acetic acid, the mixture was heated under reflux with magnetic stirring for 30 min. The corresponding aldehyde derivatives (1 mmol) were then added, and the reaction continued under reflux with stirring. Reaction progress was regularly monitored with TLC. After completion of the reaction, the mixture was cooled, and column chromatography was used to dry and purify the precipitate after it was diluted with 100 millilitres of cold water and collected by vacuum filtration to obtain the pure compounds F5, F6 and F7.

2-(Benzylideneamino)-3′,6′-dihydroxyspiro[isoindolin-3-one-1,9′-xanthene] F5 had a yield of 82.9%; m. p.: 188–190 °C, brown. F. r. (Ac : Hex) (3 : 2) = 0.50; ^1^H NMR (500 MHz, DMSO-d6) *δ* 9.84 (s, 2H), 9.02 (s, 1H), 7.91–7.85 (m, 1H), 7.58 (dtd, *J* = 21.4, 7.4, 1.2 Hz, 2H), 7.38 (dd, *J* = 6.8, 3.0 Hz, 2H), 7.31 (dd, *J* = 5.1, 1.9 Hz, 3H), 7.10 (dd, *J* = 7.4, 1.2 Hz, 1H), 6.62 (d, *J* = 2.3 Hz, 2H), 6.46 (d, *J* = 8.6 Hz, 2H), 6.42 (dd, *J* = 8.6, 2.4 Hz, 2H); ^13^C NMR (500 MHz, DMSO-d6) *δ* 164.17, 159.13, 152.81, 150.91, 149.80, 135.04, 134.52, 130.89, 129.66, 129.65, 129.36, 128.54, 127.29, 124.36, 123.73, 112.85, 110.75, 103.01, 65.93; HRMS (*m*/*z*): calculated for C_27_H_18_N_2_O_4_ 435.12666, found 435.12768.

2-((4-Methoxybenzylidene)amino)-3′,6′-dihydroxyspiro[isoindolin-3-one-1,9′-xanthene] F6 had a yield of 85%; m. p: 192–194 °C, light yellow, f. r. (Ac : Hex) (3 : 2) = 0.60; ^1^H NMR (500 MHz, DMSO-d6) *δ* 9.92–9.75 (m, 2H), 8.99 (s, 1H), 7.89–7.84 (m, 1H), 7.57 (dtd, *J* = 19.0, 7.3, 1.2 Hz, 2H), 7.35–7.32 (m, 2H), 7.08 (d, *J* = 7.1 Hz, 1H), 6.88 (d, *J* = 1.9 Hz, 1H), 6.62 (d, *J* = 2.2 Hz, 2H), 6.47–6.40 (m, 4H), 5.71 (s, 1H), 3.70 (s, 3H); ^13^C NMR (500 MHz, DMSO-d6) *δ* 161.64, 159.07, 150.33, 134.29, 129.59, 128.97, 128.56, 124.30, 123.60, 114.87, 112.79, 102.97, 55.82; HRMS (*m*/*z*): calculated for C_28_H_20_N_2_O_5_ 465.14722, found 465.14145.

2-((4-Nitrobenzylidene)amino)-3′,6′-dihydroxyspiro[isoindolin-3-one-1,9′-xanthene] F7 had a yield of 90.5%; m. p: 180–182 °C, yellow. F. r. (Ac : Hex) (3 : 2) = 0.66; ^1^H NMR (500 MHz, DMSO-d6) *δ* 10.02 (d, *J* = 112.9 Hz, 2H), 9.11 (s, 1H), 8.16 (d, *J* = 8.6 Hz, 2H), 7.91 (d, *J* = 7.5 Hz, 1H), 7.64 (dd, *J* = 7.9, 5.2 Hz, 3H), 7.58 (t, *J* = 7.4 Hz, 1H), 7.12 (d, *J* = 7.6 Hz, 1H), 6.64 (d, *J* = 2.3 Hz, 2H), 6.47 (d, *J* = 8.6 Hz, 2H), 6.42 (dd, *J* = 8.7, 2.4 Hz, 2H); ^13^C NMR (500 MHz, DMSO-d6) *δ* 164.56, 159.27, 152.77, 150.98, 148.54, 146.54, 141.20, 134.99, 129.82, 129.09, 128.50, 128.14, 124.65, 124.47, 123.97, 112.99, 110.44, 103.11, 66.14.; HRMS (*m*/*z*): calculated for C_27_H_17_N_3_O_6_ 480.11174, found 480.11212.

#### 2.2.4. Synthesis of target compounds F2–F4

Two convergent synthetic methods were developed to synthesize the xanthene-hydrazone derivatives F2–F4. In the first method some drops of glacial acetic acid were added to a round-bottom flask containing one equivalent of chemical F1 dissolved in ethanol then heated and refluxed to completely dissolve the precursor F1. TLC was used to monitor the reaction; thereafter, one equivalent of the benzaldehyde derivatives was added until all the original reagents had disappeared. Once the mixture cooled, addition of cold water induced precipitation to develop. Filtration was used to extract the xanthene hydrazone derivatives F2, F3 and F4, which were subsequently purified using column chromatography.

For the second method, a flask containing one equivalent of the F5 and F7 derivatives was completely dissolved in DMF. Then 2.2 equivalents of K_2_CO_3_ were added, and a catalytic amount of BTBA (phase transfer catalyst) was added. At room temperature, the reaction mixture was stirred for thirty minutes. 2.2 equivalents of ethyl bromoacetate were then added to the mixture after it had been stirred until the desired xanthene hydrazone derivatives F2, F3 and F4 were generated. The mixture was added to cold water after the reaction, and the precipitate that formed was filtered off. The crude material was further purified by column chromatography using an appropriate solvent mixture as eluent to afford the pure products F2, F3 and F4.

The yield of diethyl 2,2′-((2-((4-nitrobenzylidene)amino)spiro[isoindolin-3-one-1,9′-xanthene]-3′,6′-diyl)bis(oxy))(*E*)-diacetate F2 was 93%, light yellow, m. p.: 151–153 °C, f. r. = 0.40; ^1^H NMR (500 MHz, DMSO-d6): *δ* 9.33 (s, 1H), 8.23–8.17 (m, 1H), 8.13 (ddd, *J* = 8.2, 2.4, 1.1 Hz, 1H), 7.98–7.87 (m, 1H), 7.79 (dt, *J* = 7.9, 1.3 Hz, 1H), 7.67–7.57 (m, 2H), 7.17 (dt, *J* = 7.8, 0.9 Hz, 1H), 6.88 (t, *J* = 1.5 Hz, 2H), 6.63 (d, *J* = 1.5 Hz, 5H), 4.79 (s, 4H), 4.11 (q, *J* = 7.1 Hz, 4H), 1.13 (t, *J* = 7.1 Hz, 6H). ^13^C NMR (126 MHz, DMSO-d6): *δ* 168.92, 164.39, 159.24, 152.81, 150.18, 148.74, 148.12, 136.83, 134.98, 133.30, 130.95, 130.09, 128.65, 125.11, 124.51, 124.12, 121.43, 112.95, 112.46, 102.52, 65.96, 65.45, 61.23, 40.33, 14.49. HRMS (*m*/*z*): calculated for C_35_H_29_N_3_O_10_ 652.18529, found 652.18378 [M + H]^+^.

The yield of diethyl 2,2′-((2-(benzylideneamino)spiro[isoindoline-3-one-1,9′-xanthene]-3′,6′-diyl)bis(oxy))(*E*)-diacetate F3 was 78%, light yellow, m. p: 123–125 °C, f. r. = 0.37; ^1^H NMR (500 MHz, DMSO-d6): *δ* 9.21 (s, 1H), 7.93–7.88 (m, 1H), 7.65–7.56 (m, 2H), 7.40–7.33 (m, 2H), 7.31 (d, *J* = 7.3 Hz, 3H), 7.13 (d, *J* = 7.1 Hz, 1H), 6.86 (d, *J* = 1.9 Hz, 2H), 6.61 (d, *J* = 2.0 Hz, 4H), 4.79 (s, 4H), 4.12 (q, *J* = 7.1 Hz, 4H), 1.14 (t, *J* = 7.1 Hz, 6H); ^13^C NMR (126 MHz, DMSO-d6): *δ* 168.95, 164.14, 159.14, 152.72, 151.11, 150.29, 134.93, 134.66, 131.03, 129.97, 129.82, 129.33, 128.74, 127.35, 124.42, 123.92, 113.08, 112.40, 102.43, 65.43, 61.23, 14.53; HRMS (*m*/*z*): calculated for C_35_H_30_N_2_O_8_ 607.20022, found 607.20333.

The yield of diethyl 2,2′-((2-((4-methoxybenzylidene)amino)spiro[isoindolin-3-one-1,9′-xanthene]-3′,6′-diyl)bis(oxy))(*E*)-diacetate F4 was 83.5%, white, m. p.: 136–138 °C, f. r. = 0.37; ^1^H NMR (500 MHz, DMSO-d6): *δ* 9.16 (s, 1H), 7.93–7.85 (m, 1H), 7.64–7.54 (m, 2H), 7.36–7.29 (m, 2H), 7.14–7.09 (m, 1H), 6.90–6.83 (m, 4H), 6.64–6.55 (m, 4H), 4.79 (s, 4H), 4.12 (q, *J* = 7.1 Hz, 4H), 3.70 (s, 3H), 1.14 (t, *J* = 7.1 Hz, 6H); ^13^C NMR (126 MHz, DMSO-d6): *δ* 168.97, 163.90, 161.73, 159.07, 152.71, 151.61, 150.26, 130.03, 129.00, 128.75, 127.46, 113.16, 112.33, 65.71, 65.39, 61.24, 14.54; HRMS (*m*/*z*): calculated for C_36_H_32_N_2_O_9_ 637, 21078, found 636, 21326.

### 2.3. Biological activity

#### 2.3.1. Chemical reagents and medicines

Acetylcholine chloride, epinephrine, and verapamil were procured from Sigma Chemical Co. (St. Louis, USA).

#### 2.3.2. Animals

The Missour Experimental Centre in Morocco provided healthy adult male albino Wistar rats, weighing between 150 and 250 g. These animals were individually housed in plastic cages under standard laboratory conditions. They were fed a standard pellet diet tailored to their nutritional needs. An acclimatization period of at least three weeks was allowed to minimize transport-related stress and ensure their well-being before the start of the experiments. All animal procedures were carried out in accordance with the guidelines for the care and use of laboratory animals and were approved by the local ethical committee (AREC-FSTE-12/2020).

#### 2.3.3. Measurement of vascular relaxation effect mediated by the synthesized congeners

Thoracic aortas were isolated from six male rats anesthetized with sodium pentobarbital prior to euthanasia. Immediately after dissection, the aortas were placed in cold physiological buffer to preserve tissue integrity. The vessels were carefully cleaned of adhering fat and connective tissue and then cut into rings of 3–4 mm in length. Each aorta yielded two rings, resulting in a total of 12 rings. The rings were suspended under a resting tension of 2 g in a 40 mL organ bath containing Krebs–Henseleit solution, continuously oxygenated and maintained at 37 °C with a pH of 7.4. For each experimental condition (FH, F1–F7, and verapamil), 3–4 independent experiments were performed using rings derived from different animals to ensure biological reproducibility. The Krebs–Henseleit solution had the following composition: NaCl (118 mM), KCl (4.5 mM), NaHCO_3_ (25 mM), MgSO_4_ (1.2 mM), CaCl_2_ (1.8 mM), NaHPO_4_ (1.2 mM) and glucose (11 mM). To measure force variations, we used an Erma lever transducer. Each aortic ring was secured between two hooks made of stainless steel: one connected to the base of the chamber, the other connected to a UF1 force transducer, which was in turn linked to data acquisition system (PowerLab/400). This allowed us to accurately record isometric tension. We verified the integrity of the endothelium by observing a 40–60% relaxation of the acetylcholine-precontracted rings after epinephrine stimulation. After one hour of equilibration under an initial tension of 2 g, the rings were induced to contract by treating them with 10 µM epinephrine to evaluate their contractile response. They were then rinsed with Krebs solution, three times, to restore their baseline tension, and the medium was changed every 1 h.

To measure the relaxation capacity of a compound, we tested different concentrations. After an equilibration phase, the aortic rings were contracted with epinephrine (10 µM). Once the contraction had stabilized, we gradually added increasing concentrations of the compound (12.5, 25, 37.5 and 50 µg mL^−1^), waiting 10 minutes between each addition for the response to stabilize. The compounds were solubilized in 1% DMSO. The DMSO concentrations used as controls were 0.0025%, 0.005%, 0.0075% and 0.01%. Control tests confirmed that the solvent had no significant effect on the vascular tone of the rings. Relaxation was expressed as a percentage of epinephrine-induced contraction, following a protocol already described in the literature.^28^

### 2.4. *In silico* studies

#### 2.4.1. Molecular docking studies

Docking studies were conducted to gain insight into the potential binding mode of the new compounds into the active site of CaV1.2, which is a human L-type calcium channel. The 3D structure of CaV1.2 (PDB ID: 8HMB) was obtained from the RCSB Protein Data Bank.^29^ Protein preparation was performed using Swiss-PdbViewer,^30^ which included the following: eliminating water molecules and heteroatoms that were part of the crystal, adding missing hydrogen atoms, and optimizing the structure to guarantee correct geometry and stability. The ligand structures previously optimized using MMFF94 force field at Avogadro, were converted into PDBQT format before being docked. The molecular docking simulations were then carried out by use of AutoDock Vina,^31^ a widely used program for predicting ligand–protein binding conformations. A cubic grid box with dimensions of 15 Å × 15 Å × 15 Å and a spacing of 0.375 Å was defined, centred on the active site of CaV1.2 at coordinates (*X* = 167.02; *Y* = 164.68; *Z* = 154.11). Both the visualization and analysis of the resulting complexes were conducted by use of BIOVIA Discovery Studio after the completion of the docking simulations.^32^

#### 2.4.2. Molecular dynamics (MD) simulations

To assess the stability of the protein–ligand complexes and the structural fluctuations of the ligands, MD simulations were carried out on the selected compounds F3 and F4 and the reference drug verapamil. The Desmond v3.6 package was used along with the OPLS3e force field for a total simulation period of 100 ns.^33–35^ Desmond's System Builder configured a cubic water box with the TIP3P water model and a 10 Å buffer. Counterions (Na^+^ and Cl^−^) were added to guarantee electrostatic equilibrium. With a thermostat relaxation time of 1 ns, the simulation was conducted at a constant temperature of 300 K and a pressure of 1 atm. The Nose–Hoover chain thermostat and the Martyna–Tobias–Klein “barostat” techniques were used to control the temperature and pressure. A 100 ns production simulation was run under NPT ensemble conditions following equilibration. Protein stability and important interactions between ligands and critical residues were evaluated by looking at stability measures such as radius of gyration (RGyr), ligand–protein interactions, and root mean square deviation (RMSD) and root mean square fluctuation (RMSF).^36^

#### 2.4.3. ADME-Tox predictions

The overall safety of the synthesized compounds was assessed by evaluating their Absorption, Distribution, Metabolism, Excretion, and Toxicity (ADME-Tox) properties. ADME-Tox properties were evaluated using computational methods commonly used in the early stages of drug discovery to assess the suitability of small molecules as drug candidates.^36^ The pkCSM web server was employed to determine the attributes of absorption, distribution, metabolism, excretion, and toxicity.^37^

### 2.5. Statistical analysis

Data are presented as mean ± standard deviation. The Shapiro–Wilks test was used to guarantee normality, and Levene's test was used to assess the assumption of homogeneity of variance. Two-way ANOVA and Bonferroni's post hoc test for multiple comparisons were used for statistical analysis (Prism 8, GraphPad). A *P*-value of less than 0.05 was considered statistically significant.

## 3. Results and discussion

### 3.1. Chemistry

**Scheme 1:**
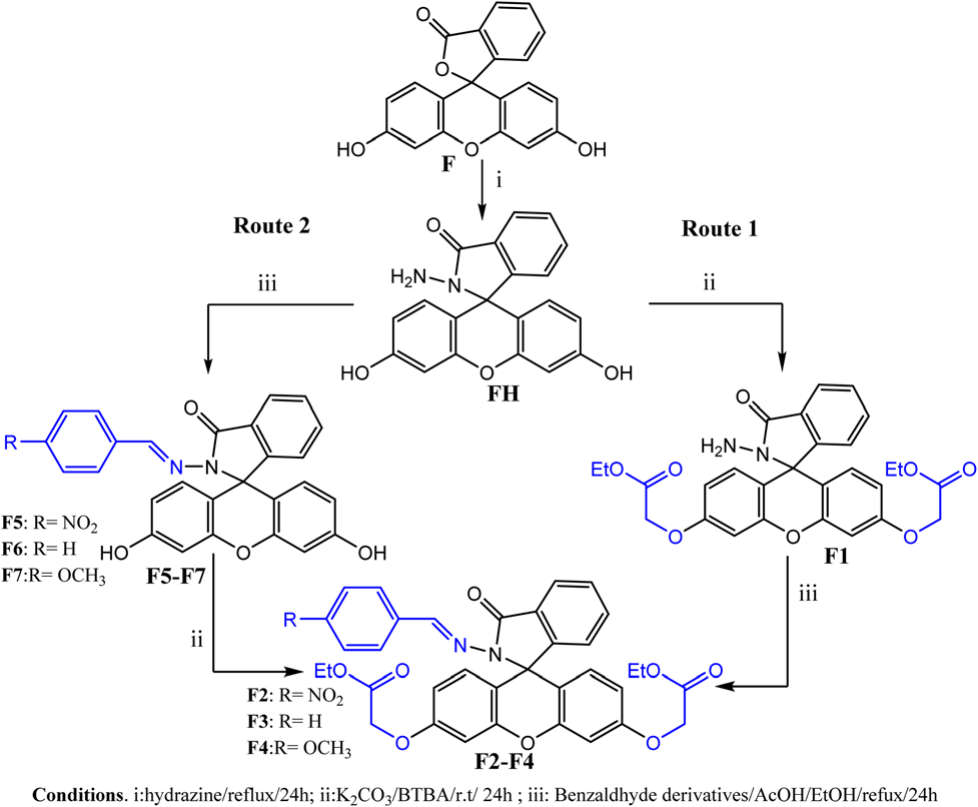
Sequential synthesis of functionalized xanthene-hydrazone derivatives *via* two protocols.

The novel functionalized xanthene-hydrazone derivatives were synthesized using two distinct methodological approaches (Scheme 1). The first protocol is based on sequential functionalization, beginning with *O*-alkylation carried out by phase transfer catalysis, followed by functionalization at the nitrogen (N) position to form the hydrazone function. This last step was carried out under reflux in an acidic medium using a conventional method. In the second protocol, these steps are applied in reverse order, *i.e.*, *N*-functionalization was carried out first, followed by *O*-alkylation.

The structures of intermediates FH, F1, and F5 through 7, as well as that of the newly synthesized target molecules F2, F3 and F4 were checked using various spectroscopic techniques and mass spectrometry. The NMR results for compound F4 are presented as a representative example of this series. In the ^1^H NMR spectrum, a singlet is observed, corresponding to the six protons of the terminal methyl groups (–CH_3_) of the ethoxy chains. Another singlet at 4.79 ppm is assigned to the four protons of the two O–CH_2_ groups bonded to the aromatic ring, while a signal at 4.12 ppm corresponds to the O–CH_2_ protons associated with the ester function. A singlet at 3.70 ppm is assigned to the three protons of the methoxy group carried by the benzene ring. In addition, a singlet signal at 9.16 ppm is characteristic of the proton of the imine function (N=CH). Finally, the signals between 6.5 and 8 ppm are assigned to the aromatic protons. The ^13^C NMR spectrum of compound F4 shows four main signals at 65.39 ppm, 61.24 ppm, 55.78 ppm, and 14.54 ppm, corresponding respectively to the carbons of the O–CH_2_ methylene groups bonded to the aromatic ring, the ester methylene (CO–O–CH_2_), the C–CH_3_ carbon of the ethoxy group, and the methoxy group of the benzene ring. An additional signal at 65.71 ppm is attributed to the spiro carbon characteristic of the spiro-lactam form of fluorescein. The most relevant NMR signals for compound F4 are shown in Table 1.

**Table 1:** Key ^1^H and ^13^C NMR spectroscopic data for compound F4

Compound F4	No.	^1^H NMR (*δ* ppm)	^13^C NMR (*δ* ppm)
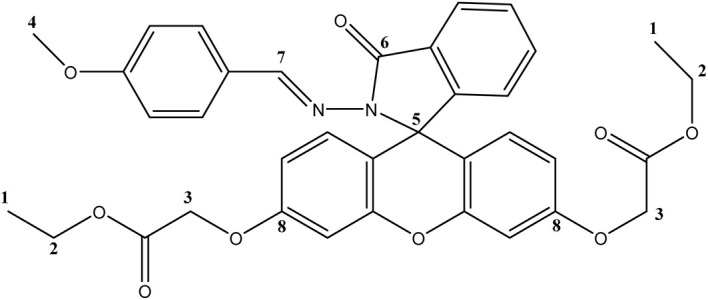	1	1.14	14.54
	2	4.12	61.24
	3	4.79	65.39
	4	3.70	55.78
	5	—	65.71
	6	—	168.97
	7	9.16	161.73
	8	—	163.90

High-resolution mass spectrometry of F4 revealed the pseudomolecular ion [M + H]^+^ at *m*/*z* 637.21328, consistent with the expected molecular mass. All NMR and HRMS spectra of the other synthesized compounds are given in the SI section.

### 3.2. Vasorelaxant activity

**Fig. 3:**
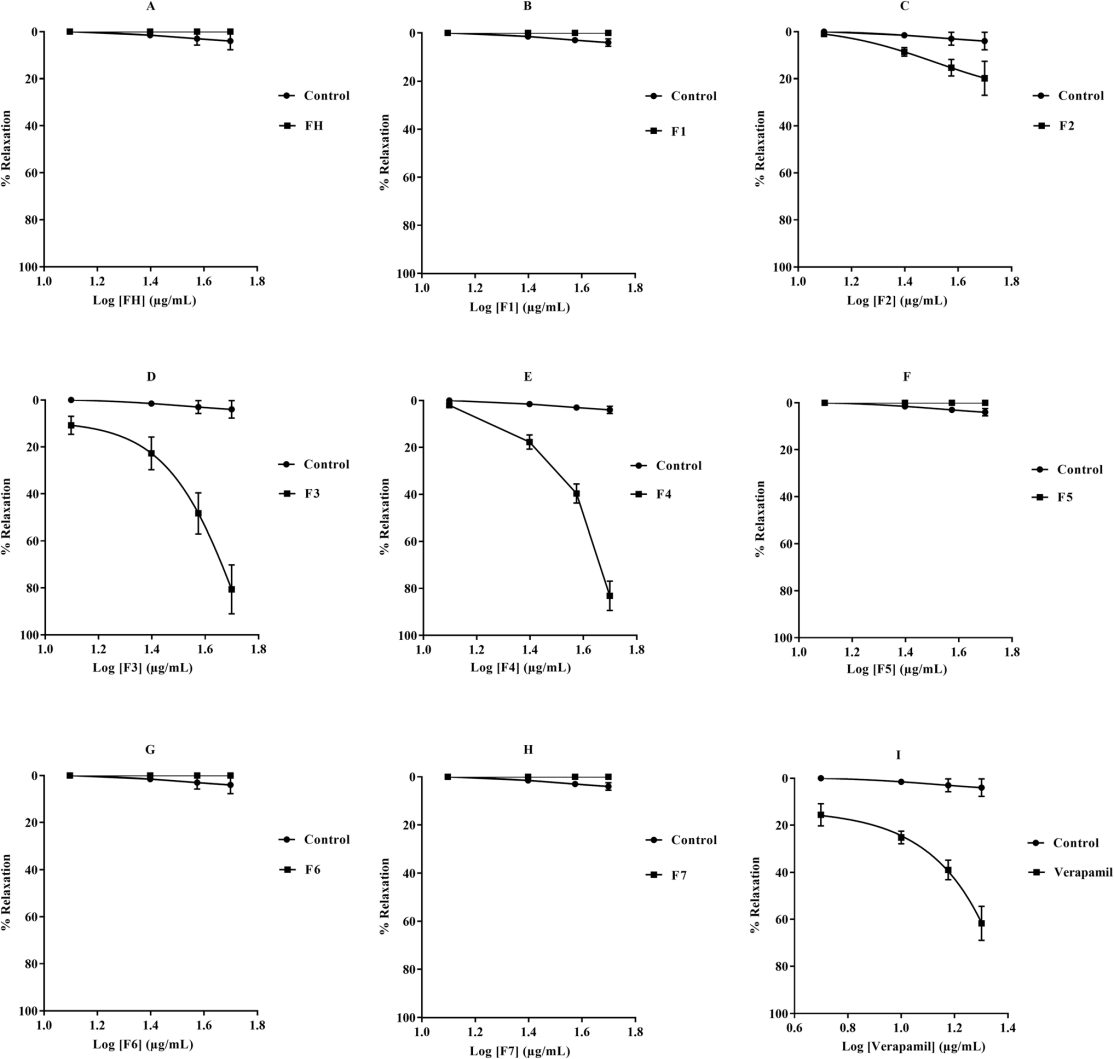
Dose–response curves illustrating the vasorelaxant effects of cumulative concentrations in rat aortic rings precontracted with EP (10 µM). Panels show: (A) FH, (B) F1, (C) F2, (D) F3, (E) F4, (F) F5, (G) F6, (H) F7, and (I) verapamil (positive control). Data are presented as mean ± SEM; *p < 0.05, **p < 0.01, ***p < 0.001, ****p < 0.0001 *vs.* control.

We conducted the evaluation in several stages. First, we tested the vasorelaxant effect of fluorescein hydrazine (FH) itself. Next, we examined the impact of alkylation on the hydroxyl group (OH), particularly for compound F1. Then, we studied separately the effect of hydrazone formation on the amine function (NH_2_), which corresponds to compounds F5 to F7. Finally, we analysed the combined influence of *O*-alkylation and hydrazone formation on compounds F2, F3 and F4. This allowed us to evaluate the synergistic role of these chemical modifications on vasorelaxant activity. The results are given in Fig. 3 and Table 2.

**Table 2:** EC_50_ and *E*_max_ values of synthesized compounds (FH–F7) and verapamil for vasorelaxant activity on rat aortic rings precontracted with epinephrine (10 µM)

Compounds	EC_50_ (µg mL^−1^)	*E*_max_ (%)
FH	0.000 ± 0.000	00.00 ± 0.00
F1	0.000 ± 0.000	00.00 ± 0.00
F2	113.000 ± 8.00	19,75 ± 4.10
F3	38.204 ± 5.00	80.61 ± 6.00
F4	41.300 ± 5.000	83.13 ± 6.20
F5	0.000 ± 0.000	00.00 ± 0.00
F6	0.000 ± 0.000	00.00 ± 0.00
F7	0.000 ± 0.000	00.00 ± 0.00
Verapamil	18.000 ± 1.000	61.67 ± 4.10

The results show that fluorescein hydrazine (FH) does not exhibit significant vasorelaxant activity. This lack of efficacy suggests that its chemical structure, as designed, does not interact effectively with the membrane receptors or signalling pathways involved in vascular relaxation. Furthermore, derivatives modified at the O position, such as product F1, were also found to be inactive. This indicates that simple substitution on the hydroxyl group is not sufficient to confer pharmacological activity. Evaluation of *N*-functionalization, corresponding to the formation of the hydrazone function, showed that *N*-functionalized compounds (F5–F7) exert no significant vasorelaxant activity, with EC_50_ and *E*_max_ parameters remaining zero under our experimental conditions.

Double functionalization, combining modifications at the O and N positions, aimed to simultaneously modulate the polarity, lipophilicity and electron density of the fluorescein nucleus to optimize molecular recognition at the level of vascular receptors. This strategy yielded very different results: marked vasorelaxant activity, unlike the simple derivatives, which remained inactive. The EC_50_ and *E*_max_ values for compounds F2 to F4 reveal a clear hierarchy, which is dependent on the position and nature of the aromatic substituents introduced. This confirms the extent to which the chemistry of these groups influences pharmacological activity.

Compounds F3, bearing an unsubstituted aromatic ring, and F4, substituted by a methoxy group in the para position, exhibit moderate to good vasorelaxant activity. They differ markedly from fluorescein hydrazone (FH) and its simple derivatives F1 and F5 to F7, which are inactive. Their EC_50_ values are 38.204 and 41.3 µg mL^−1^ respectively, while their *E*_max_ values (80.61% and 83.13%) indicate significant activity. This improvement suggests that a moderate increase in the electron density of the aromatic ring promotes the recognition of compounds by receptors or channels involved in vasodilation. In contrast, the F2 derivative, substituted by a nitro group (NO_2_), shows a notable reduction in activity (EC_50_ = 113 µg mL^−1^; *E*_max_ = 19.75%). This substitution disrupts conjugation and impairs interaction with the biological target.

These collective findings establish a definitive structure–activity relationship that constitutes the central discovery of this study: dual functionalization at both O- and N-positions is strictly required for vasorelaxant activity. The complete inactivity of singly modified derivatives (F1, F5–F7) *versus* the marked potency of doubly functionalized congeners (F3, F4) demonstrates that neither the ethyl ester groups alone, nor the hydrazone moiety alone can activate the underlying biological mechanism. Instead, the simultaneous presence of both pharmacophores appears necessary to achieve the optimal balance of polarity, lipophilicity, and electron density required for effective interaction with vascular receptors or ion channels.

### 3.3. Molecular docking studies

**Fig. 4:**
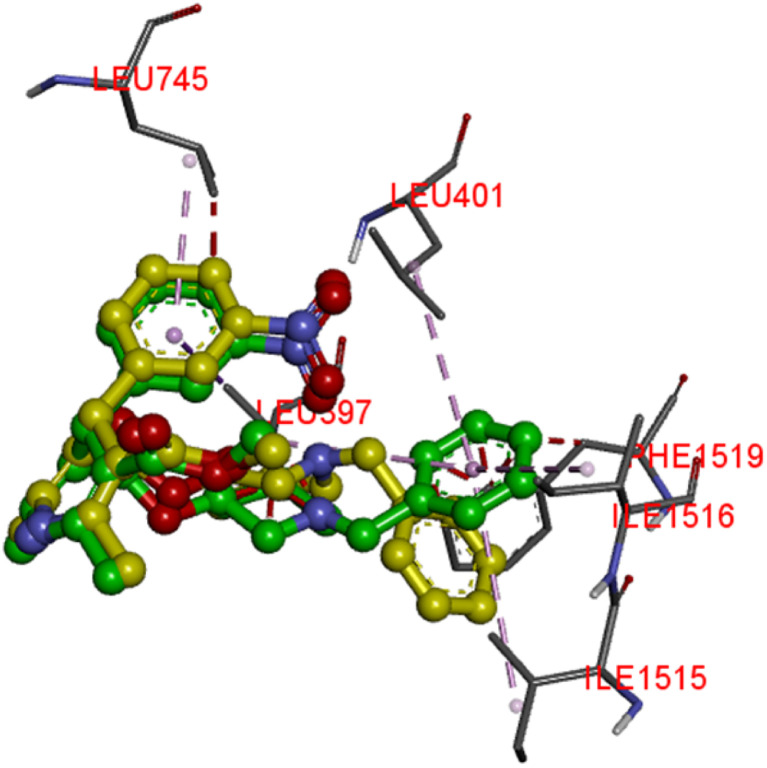
Native ligand (yellow) overlaps with its best docked pose (green) in a re-docking protocol.

To understand the mechanism of action and binding affinities of the synthesized compounds toward the human L-type calcium channel (CaV1.2), molecular docking simulations were performed. CaV1.2 was chosen as the molecular target owing to its essential role in regulating smooth muscle contraction and promoting vascular relaxation.^38^ The channel therefore represents a favourable target in the treatment of cardiovascular disorders, particularly those characterized by an excessive rate of smooth muscle contraction, since it controls the vascular tone by modulating the calcium ion influx into the cells.^39,40^ The crystal structure with PDB ID: 8HMB, co-crystallized with verapamil, was used to compare the binding affinities and interaction profiles of the synthesized derivatives with the reference drug. Before the docking simulations, re-docking of the native ligand within the protein's active site was carried out. The RMSD value of 1.27 Å resulting from this practice, which is significantly below the accepted threshold of 2.0 Å, validates the accuracy and reliability of the docking methodology used (Fig. 4).

**Fig. 5:**
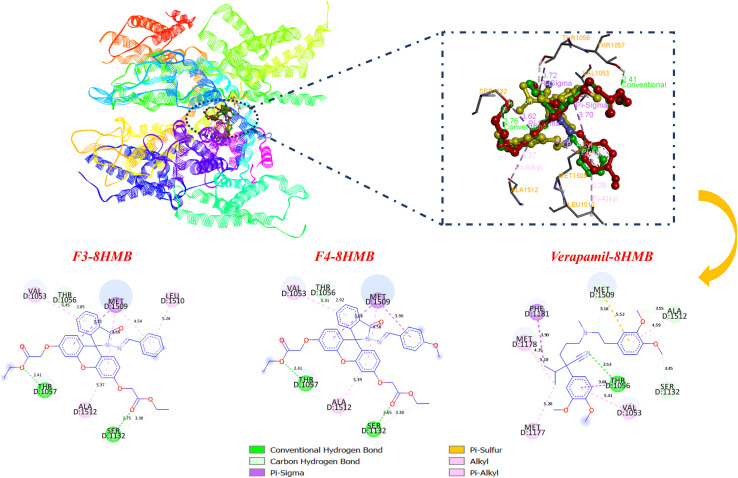
2D and 3D binding interactions of compounds F3, F4, and verapamil within the active site of CaV1.2 (PDB ID: 8HMB).

Results of docking simulations indicated that the two active compounds (F3 and F4) were all favorably situated within the CaV1.2 binding pocket, with binding energies of approximately −7.0 to −7.1 kcal mol^−1^, which were comparable to that of verapamil (−7.0 kcal mol^−1^) (Table 3). Supporting the findings of the biological tests, the compounds F3 (EC_50_ = 38.204 µg mL^−1^) and F4 (EC_50_ = 41.300 µg mL^−1^) were found to be the strongest CaV1.2 inhibitors, thus indicating their high binding affinity. 2D and 3D visual representations of the compounds F3 and F4, compared with verapamil (Fig. 5), were generated for analysis of the key interactions responsible for their enhanced affinity toward the CaV1.2 target. Verapamil exhibits a stabilizing network comprising hydrogen bonds and multiple hydrophobic contacts. Specifically, it forms a conventional hydrogen bond with Thr1056 (2.53 Å) and several carbon-hydrogen bonds with Ser1532 (3.45 Å), Met1509 (3.16 Å), and Ala1512 (3.55 Å), ensuring an optimal orientation of the ligand within the binding pocket. Additional hydrophobic interactions, including π-alkyl contacts with Val1053 (5.81 Å), Met1177 (5.20 Å), Met1178 (5.18 Å), and Ala1512 (4.59 Å), together with π-sigma interactions with Phe1181 (3.90 Å), further stabilize the complex. Compounds F3 and F4 exhibit a highly comparable interaction profile, forming conventional hydrogen bonds with Thr1057 (2.41 Å) and Ser1532 (2.75 Å), along with carbon–hydrogen bonds with Thr1056 (2.85 Å) and Ser1132 (3.30 Å). Stabilization is also reinforced by π-sigma interactions with Met1509 (3.70 Å) and π-alkyl contacts with Val1053 (5.45 Å), Ala1512 (5.37 Å), and Met1509 (4.54 Å and 4.59 Å). Notably, F3 forms an additional π-alkyl interaction that is absent in F4, probably because the methoxy substituent in F4 alters the aromatic orientation of the ligand within the binding pocket. Overall, both derivatives recapitulate almost all the important interactions observed for verapamil, taking the same polar and hydrophobic residues in the active site. This similarity in the interaction pattern explains why docking gave such close binding energies of (−7.0 to −7.1 kcal mol^−1^) and aligns with the experimental activity values. These results highlight the capability of F3 and F4 as high-affinity CaV1.2 ligands and provide a strong rationale for additional structural optimization and the development of congeners with increased activity.

**Table 3:** Binding affinity of synthesized compounds in the CaV1.2 (PDB ID: 8HMB)

Compounds	F3	F4	Verapamil
Binding affinity kcal mol^−1^	−7.1	−7.1	−7.0

### 3.4. ADME-Tox predictions

The ADMET properties of the active compounds were evaluated using the pkCSM web server to assess their pharmacokinetic potential as promising drug candidates (Table 4). Both molecules showed excellent human intestinal absorption (HIA = 100%), which is a sign of good oral bioavailability. Their Caco-2 permeability values (log *P*_app_ = 0.407 and 0.346, respectively) imply that their absorption is moderate, thus confirming their passive diffusion through the cell membranes. The volume of distribution (log *V*_Dss_) values was negative (−1.385 and −1.496 L kg^−1^, respectively), indicating limited tissue distribution, while the predicted log BB values, all less than −1.0, confirm poor penetration across the blood–brain barrier (BBB), which could potentially reduce central side effects. Regarding metabolism, both compounds are predicted to act as substrates of CYP3A4, and neither compound inhibits CYP1A2, CYP2D6, or CYP3A4, except for F4, which shows slight inhibition of CYP3A4. However, both compounds display moderate inhibitory potential toward CYP2C19 and CYP2C9, suggesting an acceptable level of metabolic interaction and maintaining overall metabolic stability. The total clearance values (0.838 and 0.824 mL min^−1^ kg^−1^, respectively) further indicate adequate excretory efficiency. Concerning toxicity predictions (Table 5), both F3 and F4 were determined to be non-sensitizing to the skin, non-hepatotoxic, and non-mutagenic (negative Ames test), reflecting an overall favourable safety profile. However, both compounds were identified as potential hERG II inhibitors, which may raise concerns regarding cardiotoxicity. As hERG channel inhibition is associated with QT interval prolongation and arrhythmias, this aspect is particularly relevant for cardiovascular agents. Nevertheless, this prediction is based solely on *in silico* models, while the present study focused on vasorelaxant activity in rat aortic rings and interaction with the CaV1.2 channel. Therefore, further experimental studies are required to confirm this potential liability and ensure cardiac safety. Overall, these *in silico* findings suggest that F3 and F4 possess acceptable ADME-Tox properties, supporting their potential as promising drug candidates.

**Table 4:** Predicted ADME properties for active derivatives

Compounds	ADME properties											
	Absorption		Distribution		Metabolism							Excretion
	Caco_2_ perm. log *P*_app_ in 10^−6^ cm s^−1^	HIA%	*V*_Dss_ (log L kg^−1^)	BBB perm. (BB log)	CYP450							Total clearance log ml min^−1^ kg^−1^
					Substrate		Inhibitor					
					2D6	3A4	1A2	2C19	2C9	2D6	3A4	
Acceptable range	Log *P*_app_ > 0.90: high	<30%: poorly absorbed	log *V*_Dss_ >0.45: high	log BB < – 1: poorly distributed	Categorical (yes/no)							—
F3	0.407	100	−1.385	−1.565	No	Yes	No	Yes	Yes	No	No	0.838
F4	0.346	100	−1.496	−1.802	No	Yes	No	Yes	Yes	No	Yes	0.824

**Table 5:** Predicted toxicity of active compounds

Compounds	Toxicity				
	AMES	Categorical (yes/no)			
		hERG I	hERG II	Hepatotoxicity	Skin Sensitization
F3	No	No	Yes	No	No
F4	No	No	Yes	No	No

### 3.5. MD simulations

**Fig. 6:**
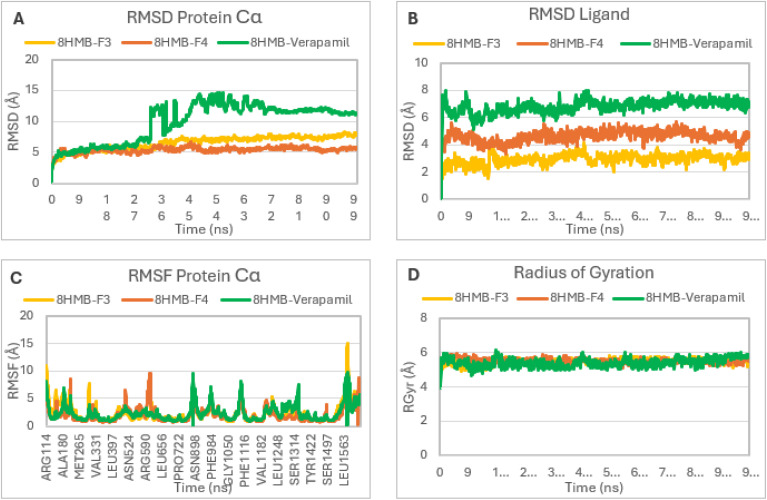
Molecular dynamics simulation trajectory analysis of the ligand–protein complexes 8HMB-F3, 8HMB-F4, and 8HMB-verapamil. (A) RMSD plot of the protein; (B) RMSD plot of the ligand; (C) RMSF plot of protein residues; (D) radius of gyration plot.

MD simulations of compounds F3 and F4, as well as verapamil, in complex with the CaV1.2 protein (PDB ID: 8HMB) were performed over a 100 ns timescale to validate the docking results and evaluate the conformational stability of the complexes. The Cα RMSD values of all systems were explored to evaluate the structural stability during the simulation. Both complexes, 8HMB-F3 and 8HMB-F4, exhibited relatively low backbone deviations ranging between 5.0 and 6.0 Å and quickly reached equilibrium after approximately 10 ns, maintaining stable trajectories for the rest of the simulation (Fig. 6A). The RMSD profile of the 8HMB-verapamil complex also showed relatively low backbone deviations (5.0–6.0 Å) during the first 30 ns, followed by pronounced fluctuations, with RMSD values increasing up to 13 Å. After about 70 ns, the system stabilized at around 11 Å, indicating significant conformational rearrangements within the complex. The average ligand RMSD values for all complexes remained consistent overall during the simulation (Fig. 6B). Specifically, compound F3 exhibited RMSD fluctuations between 2.0 and 3.5 Å, suggesting a stable orientation and minimal movement within the active site. Compound F4 displayed moderate variations ranging from 3.0 to 5.0 Å, while verapamil showed the highest ligand RMSD values (5.0–8.0 Å), indicating greater flexibility. This suggests that the F3 ligand maintained a more stable conformation and experienced fewer structural changes compared to F4 and verapamil throughout the simulation. RMSF values are also computed to establish the dynamic behavior of protein residues (Fig. 6C). The structural stability of the protein was confirmed as most residues in all systems showed a fluctuation below 5 Å. Maximum fluctuations of 15.0 Å and 10.0 Å are detected in residues Arg114, Arg590, Asn898 and Leu1563. Visual examination of the MD simulation trajectories revealed that all compounds exhibited substantial binding interactions with the hotspot residues, specifically, Gln1060, Met1178, Phe1181, Ala1512, Met1509 and Tyr1508 of the CaV1.2 protein. It was found that all these interacting residues had low RMSF values, indicating the stability of F3 and F4 complexes compared to the verapamil complex during simulation. Greater structural stability is reflected by smaller fluctuations. The radius of gyration (RGyr) was used to assess the structural compactness of complexes. The radius of gyration represents the root-mean-square distance of a molecule's atoms from its centre of mass, serving as a measure of the overall compactness and three-dimensional organisation of a macromolecule under different conditions.^41,42^ The RGyr values (Fig. 6D) for all three complexes remained nearly constant, ranging from 5.2 to 5.8 Å, throughout the 100 ns trajectory. These relatively low and favourable values suggest that the CaV1.2 protein maintained its overall structural stability throughout the simulation.

**Fig. 7:**
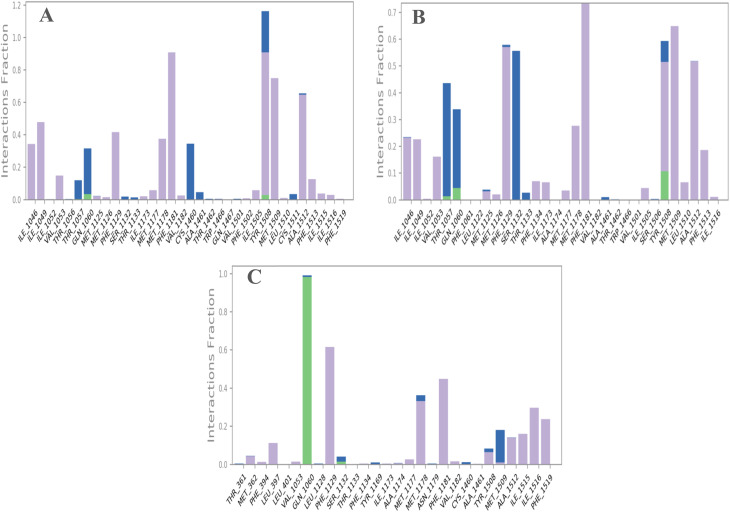
Protein–ligand interactions of complexes: (A) 8HMB-F3, (B) 8HMB-F4, and (C) 8HMB-verapamil.

Interaction analysis identified the key residues contributing to the stabilization of the complexes. The 8HMB-F3 complex (Fig. 7A) showed that the residues Tyr1508, Phe1181, and Met1509 with the highest interaction fractions of about 1.1, 0.9, and 0.7, respectively. These values reflect predominant hydrophobic and π–π interactions that persisted throughout the simulation, playing a crucial role in maintaining complex stability. The 8HMB-F4 complex (Fig. 7B) displayed a more evenly distributed interaction pattern, with interaction fractions exceeding 0.7 for Phe118, around 0.6 for Tyr1108, Met1129, and Ser1506, and between 0.4 and 0.5 for several additional residues. These interactions, having hydrophobic contacts, hydrogen bonds, and water bridges, also contributed to the overall stability of the complex. In contrast, verapamil (Fig. 7C) had an interaction with Gln1060 as the strongest one with a fraction around 1.0, along with secondary contacts with Phe1129 and Phe1181 (0.6, 0.7, respectively), reflecting the stabilization within the CaV1.2 active site. Overall, MD studies indicate that both F3 and F4 formed stable complexes with CaV1.2, with a slight preference for F3, as demonstrated by its decreased residue fluctuations and lower protein and ligand RMSD values at the active site, and maintained structural compactness. These results confirm the strong and stable binding modes of F3 and F4, supporting their potential as promising vasorelaxant agents.

## 4. Conclusions

The present study enabled the successful synthesis and characterization of a novel series of functionalized molecules (F2–F4) incorporating xanthene and hydrazone motifs using two distinct methods, combining *O*-alkylation and condensation at the NH_2_ group. *In vitro* evaluation of vasorelaxant activity on isolated rat aortic rings showed that dual functionalization, simultaneously modulating the polarity, lipophilicity and electron density of the fluorescein nucleus, optimizes molecular recognition at the level of vascular receptors. This strategy led to significant vasorelaxant activity of the hybrid molecules in the series. Molecular docking and MD analyses showed a high binding affinity and improved stability in the active site compared to verapamil, thus suggesting that these compounds are valuable candidates for vasodilator therapeutic applications. Furthermore, results of *in silico* ADMET studies revealed a good pharmacokinetic profile for compounds F3 and F4, with favourable safety profiles as evidenced by negative Ame's test results (non-mutagenic). The predicted hERG II inhibition remains the primary safety consideration requiring further experimental evaluation. Overall, these experimental and theoretical findings underscore the potential of F3 and F4 as promising vasorelaxant agents and provide a strong rationale for further structural optimization and development of congeners with enhanced activity. Further experimental studies are needed to substantiate the predicted mechanism of action.

## Conflicts of interest

Authors declare no conflict of interest.

## Supplementary Material

RA-016-D6RA01067A-s001

## Data Availability

All additional data analysed during this study can be found in the supplementary information (SI) section of this article. Supplementary information: ^1^H NMR, ^13^C NMR, and HRMS spectra of the synthesized compounds. See DOI: https://doi.org/10.1039/d6ra01067a.
