# Supplementary material for: Synthesis, characterization, in vitro and computational assessment of new xanthene hydrazone derivatives as promising vasorelaxant agents
Source: RSC Adv. 2026 Apr 17;16(22):20157–68. doi: 10.1039/d6ra01067a (PMC13090086; doi:10.1039/d6ra01067a)
Supplement: RA-016-D6RA01067A-s001 [file RA-016-D6RA01067A-s001.pdf]

Supplementary Information (SI) for RSC Advances.
This journal is © The Royal Society of Chemistry 2026



# Synthesis, Characterization, *in vitro* and Computational Assessment of New Xanthene Hydrazone Derivatives as Promising Vasorelaxant Agents

Mohammed El Mesky[1]*, Ismail bouadid[2], Fatimazahra Guerguer[3], Zgueni Hicham[1], Yassine Rhazi[4], Mohammed Chalkha[1,4]*, Farid Khallouki[2], Samir Chtita[3], Sahar Abdulaziz AlSedairy[5], Mourad A. M. Aboul-Soud[6]*, John P. Giesy[7,8,9], Driss Chebabe[1], El Houssine Mabrouk[1,4] and Mohamed Eddouks[2]

[1]*Laboratory of Materials Engineering for the Environment and Natural Resources, Faculty of Sciences and Techniques, University of Moulay Ismail of Meknès, B.P 509, Boutalamine, 52000, Errachidia, Morocco.*

[2]*Team of Ethnopharmacology and Pharmacognosy, Faculty of Sciences and Techniques Errachidia, Moulay Ismail University of Meknes, Errachidia, Morocco*

[3]*Laboratory of Analytical and Molecular Chemistry, Faculty of Sciences Ben M'Sik, Hassan II University of Casablanca, Casablanca, Morocco*

[4]*Laboratory of Engineering of Organometallic, Molecular Materials, Environment, and Innovative Pedagogy (LIMOMEPI), Faculty of Sciences Dhar EL Mahraz, Sidi Mohamed Ben Abdellah University, P.O. Box 1796 (Atlas), 30000 Fez, Morocco.*

[5] *Department of Food Sciences and Nutrition, College of Food and Agricultural Sciences, King Saud University, P.O.Box 2460, Riyadh 11451, Saudi Arabia;*

[6]*Center of Excellence in Biotechnology Research (CEBR), College of Applied Medical Sciences, King Saud University, P.O. Box 17 10219, Riyadh 11433, Saudi Arabia;*

[7]*Department of Veterinary Biomedical Sciences and Toxicology Centre, Western College of Veterinary Medicine, University of Saskatchewan, Saskatoon, SK S7N 5B4, Canada, Jgiesy@aol.com*

[8]*Department of Integrative Biology and Center for Integrative Toxicology, Michigan State University, East Lansing, MI 48824, USA*

[9]*Department of Environmental Sciences, Baylor University, Waco, 76706, USA,*

***Corresponding authors' E-mails**: *m.elmesky@edu.umi.ac.ma*; mohammed.chalkha1@usmba.ac.ma; *maboulsoud@ksu,edu.sa*

## I. *Chemical reagents and instruments*

All chemicals, solvents and reagents used were of analytical grade and used without further purification. The chemicals were purchased from commercial suppliers: Fluorescein(98.0 %), hydrazine (99.0 %), Ethanol (≥99.5 %), (99.8%), 4-methoxybenzaldhyde (98.0 %), Benzaldhyde (98.0 %), 4-nitrobenzaldhyde (98.0 %), Ethyl bromoacetate (99.8%), Dimethylformamide (99.8 %), Potassium carbonate ( ≥98.0 %), Tetrabutylammonium bromide ( ≥98.0 %),, Hexane (≥95%), Acetate d'ethyle (≥98%%).

## II. *NMR $^{1}$H Spectra of FH, F1-F7*

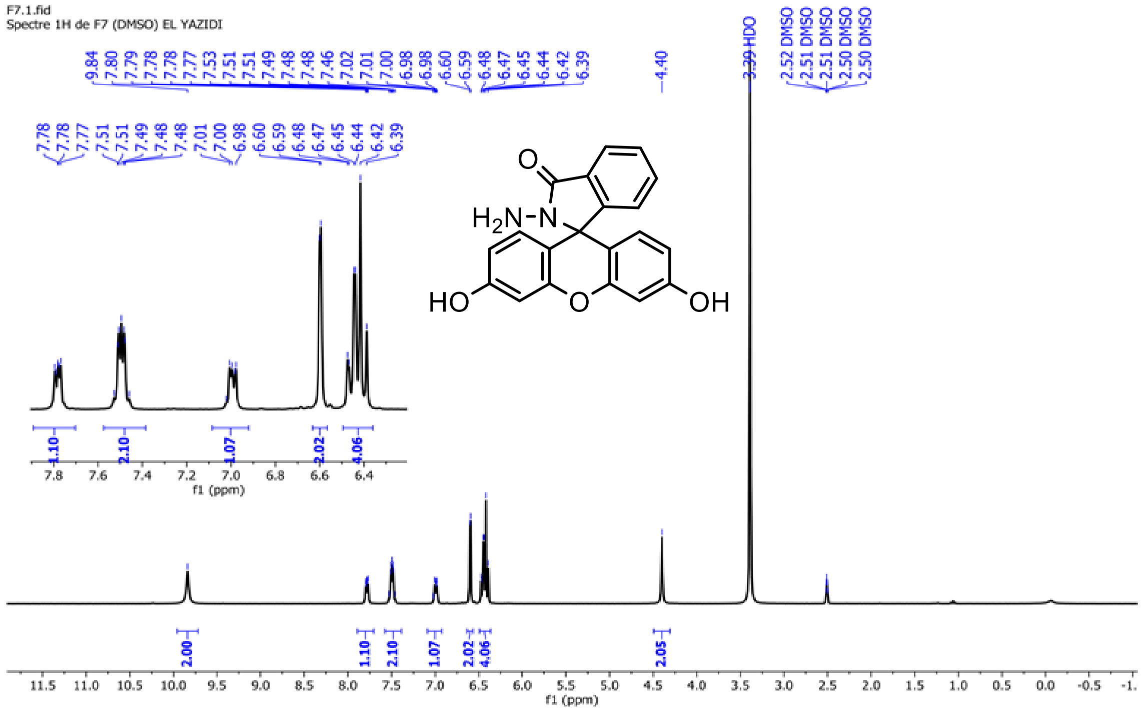


**Figure S 1. $^{1}$H NMR spectrum (300 MHz, DMSO-*d6*) of FH**

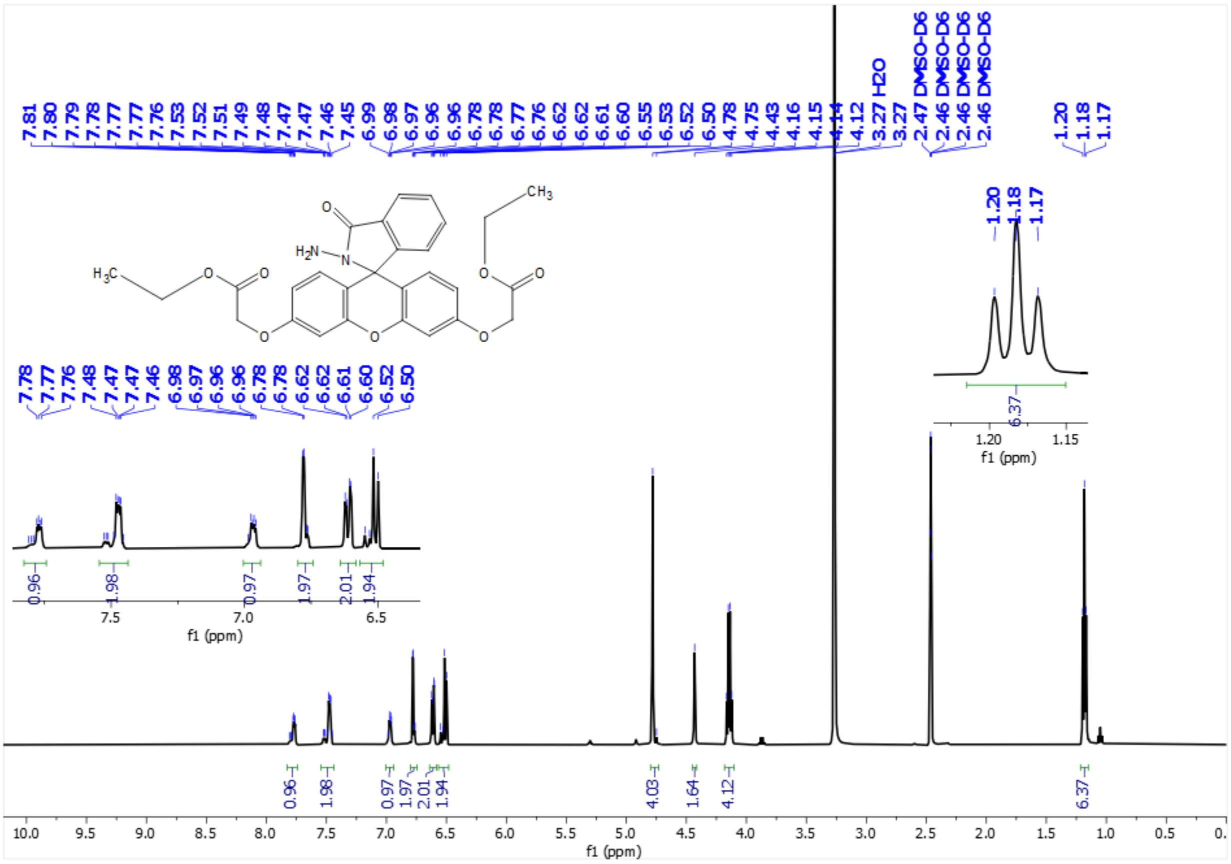


**Figure S 2. $^{1}$H NMR spectrum (500 MHz, DMSO-d6) of F1**

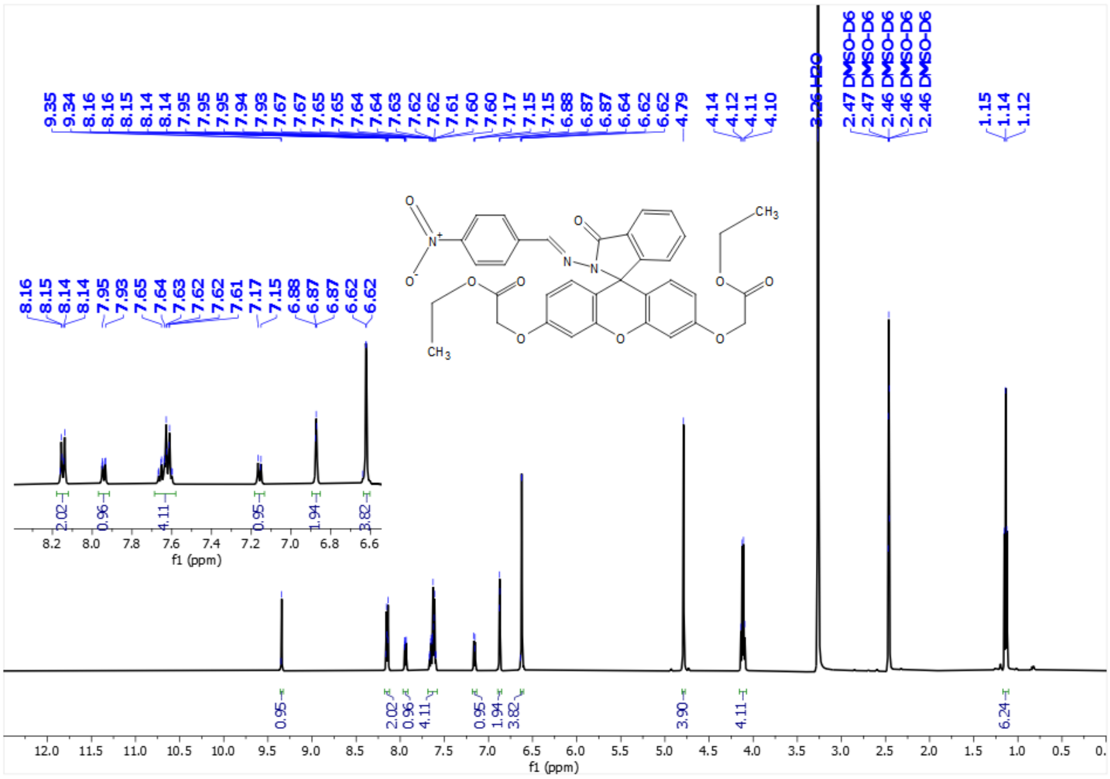


**Figure S 3. ¹H NMR spectrum (500 MHz, DMSO-d6) of F2**

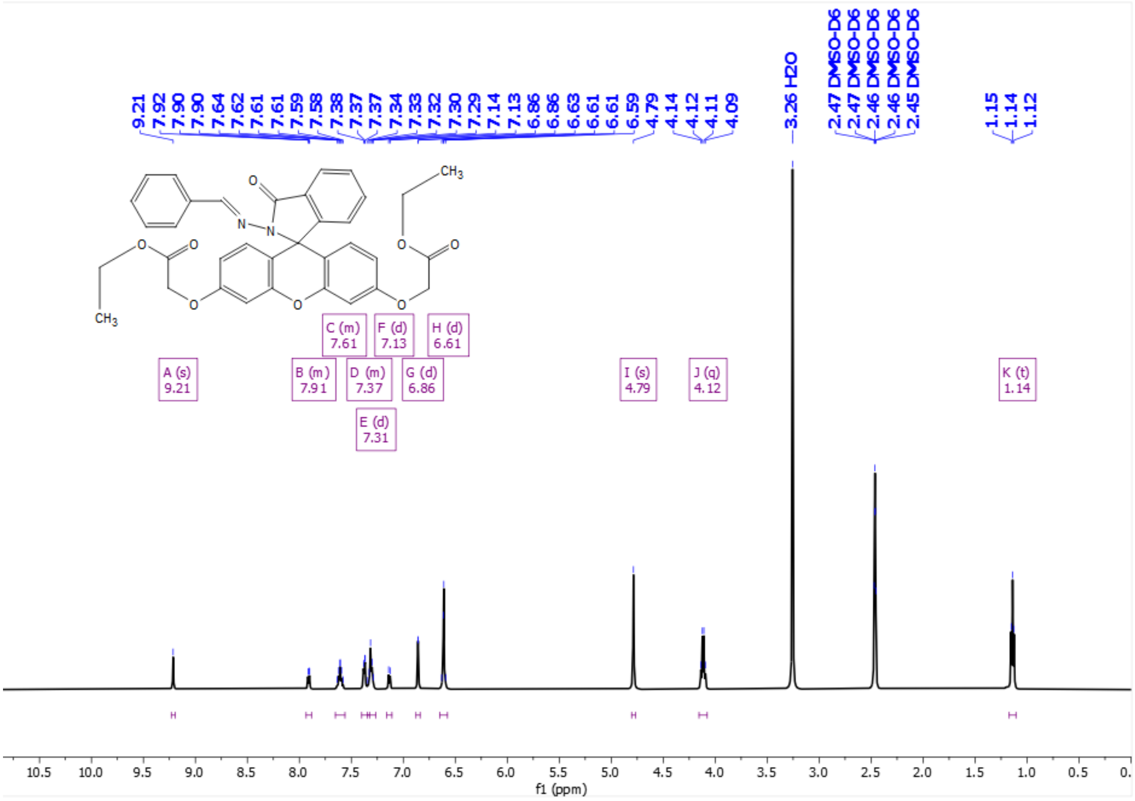


**Figure S 4. ¹H NMR spectrum (500 MHz, DMSO-d6) of F3**

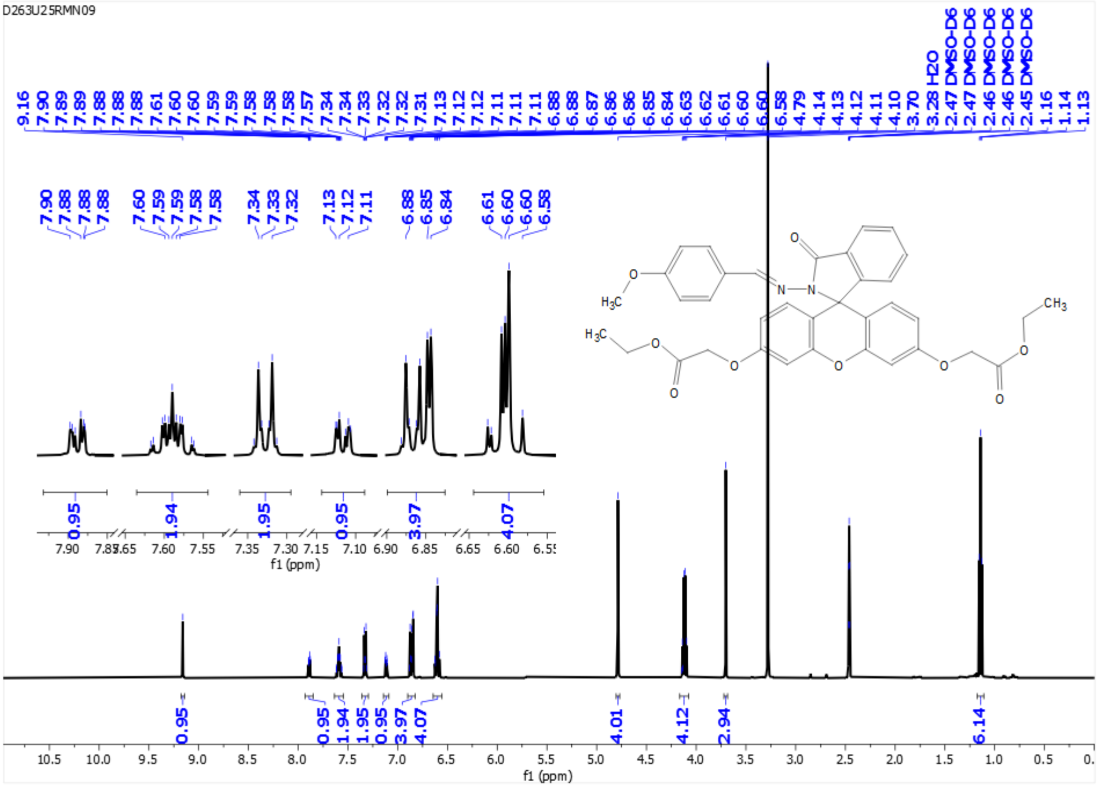


**Figure S 5. ¹H NMR spectrum (500 MHz, DMSO-d6) of F4**

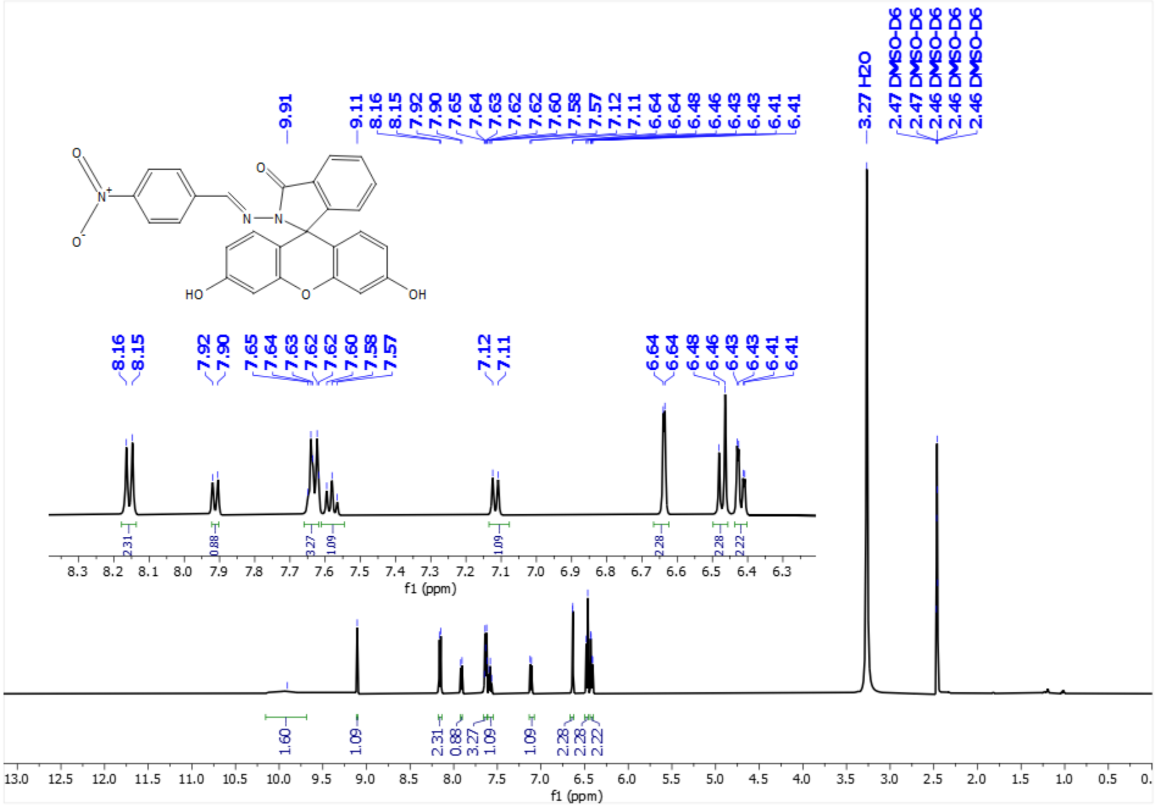


**Figure S 6. ¹H NMR spectrum (500 MHz, DMSO-d6) of F5**

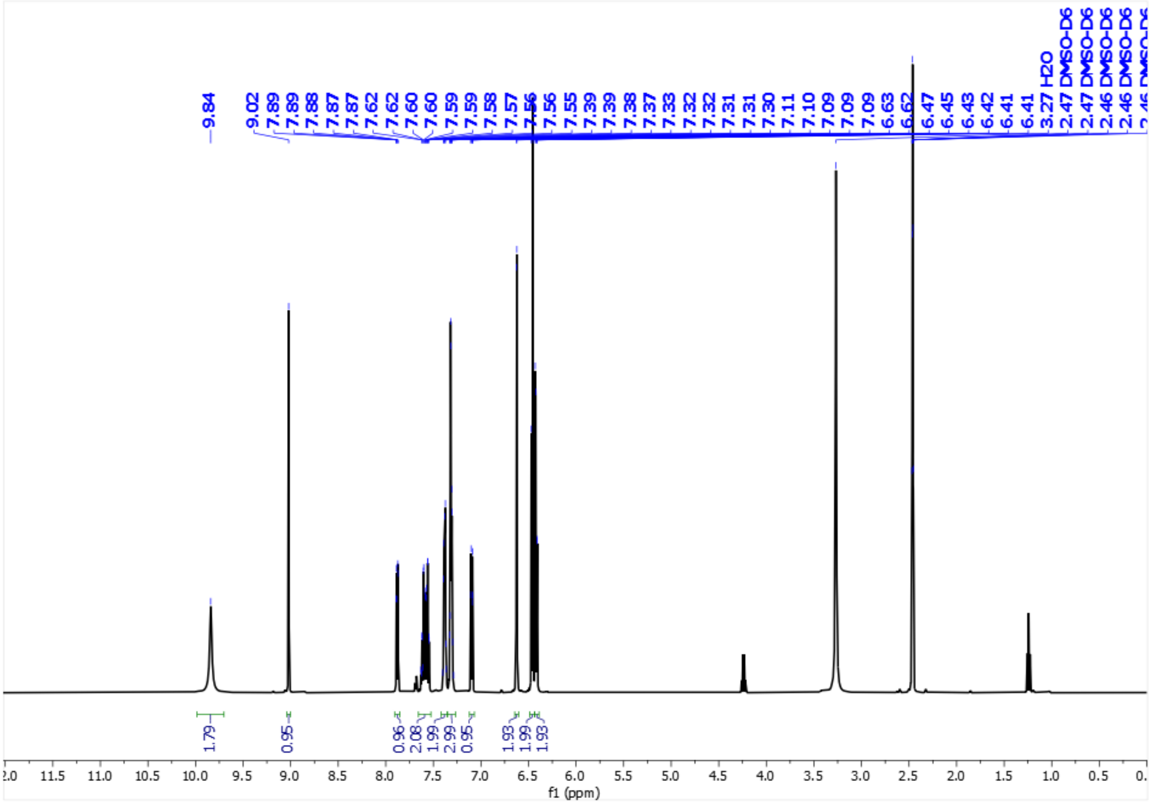


**Figure S 7. ¹H NMR spectrum (500 MHz, DMSO-d6) of F6**

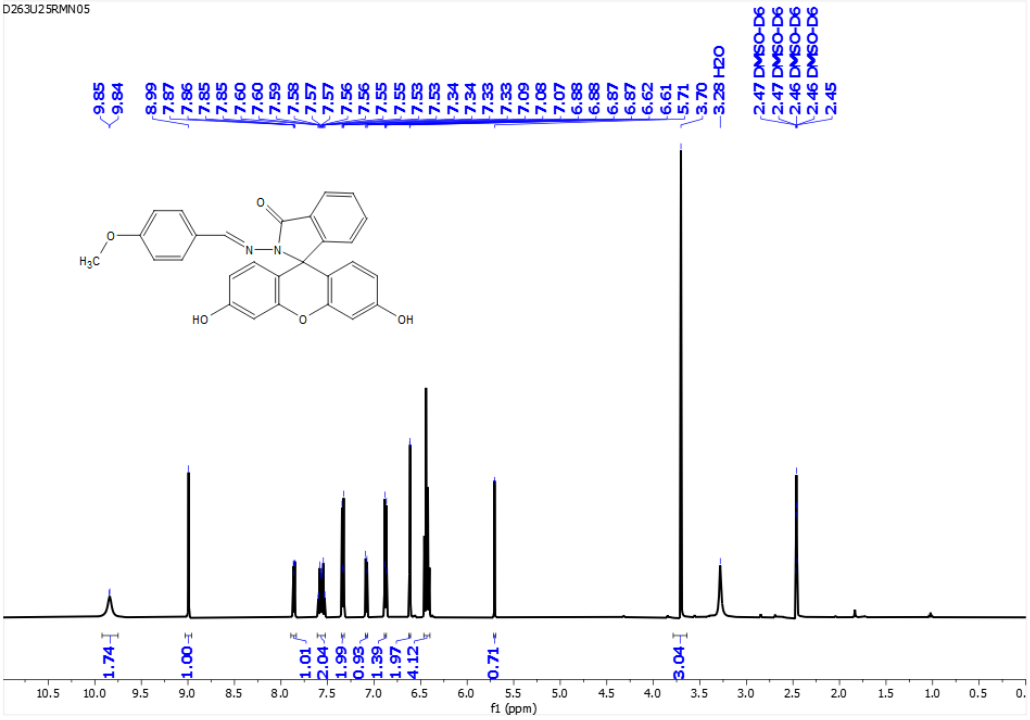


**Figure S 8. ¹H NMR spectrum (500 MHz, DMSO-d6) of F7**

## III. *NMR [13]C Spectra of FH, F1-F7*

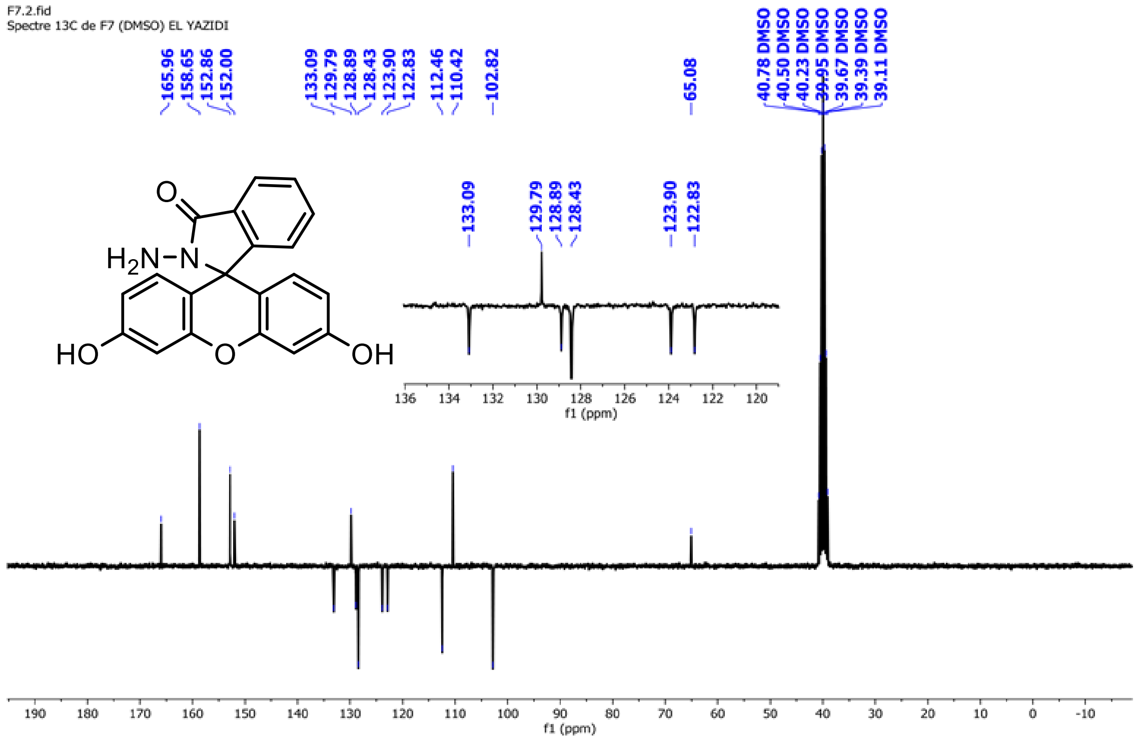


**Figure S 9. $^{13}$C NMR spectrum (300 MHz, DMSO-d6) of FH**

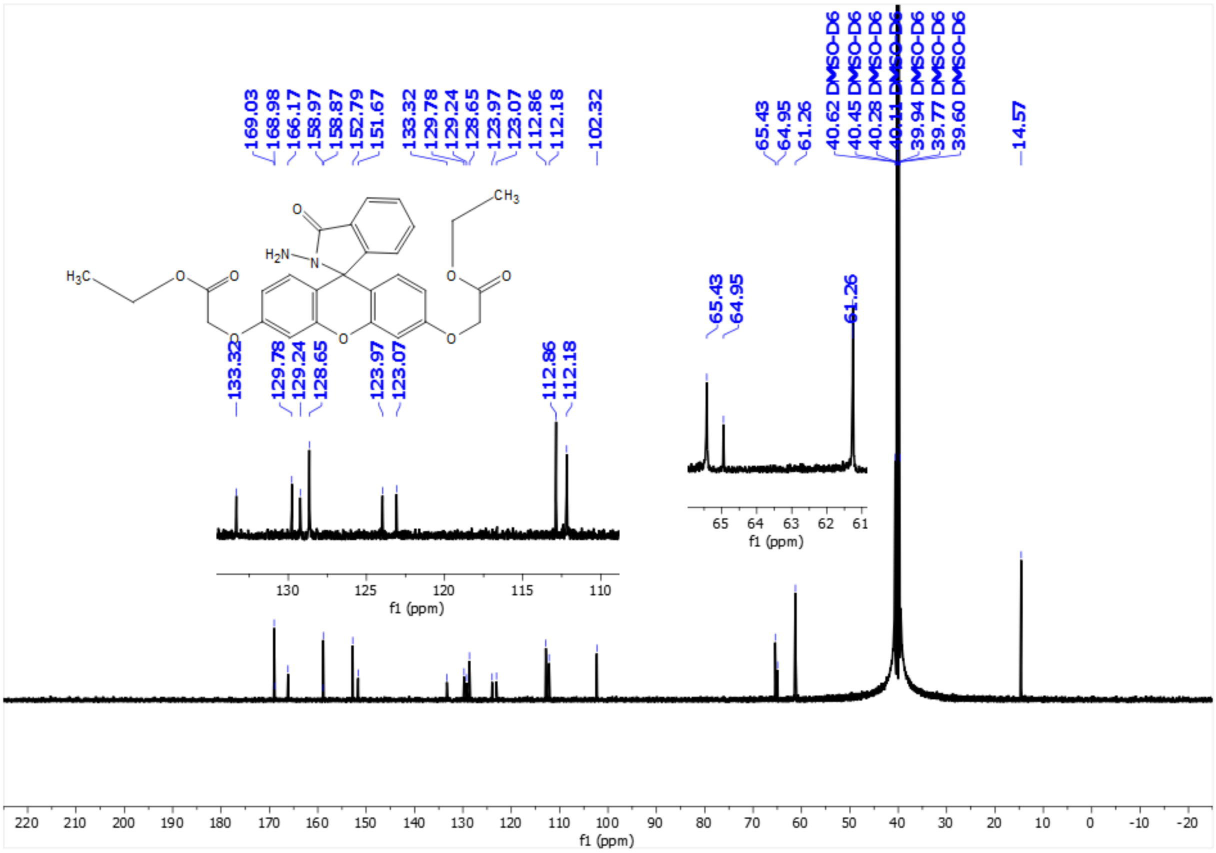


**Figure S 10. ¹3C NMR spectrum (300 MHz, DMSO-d6) of F1**

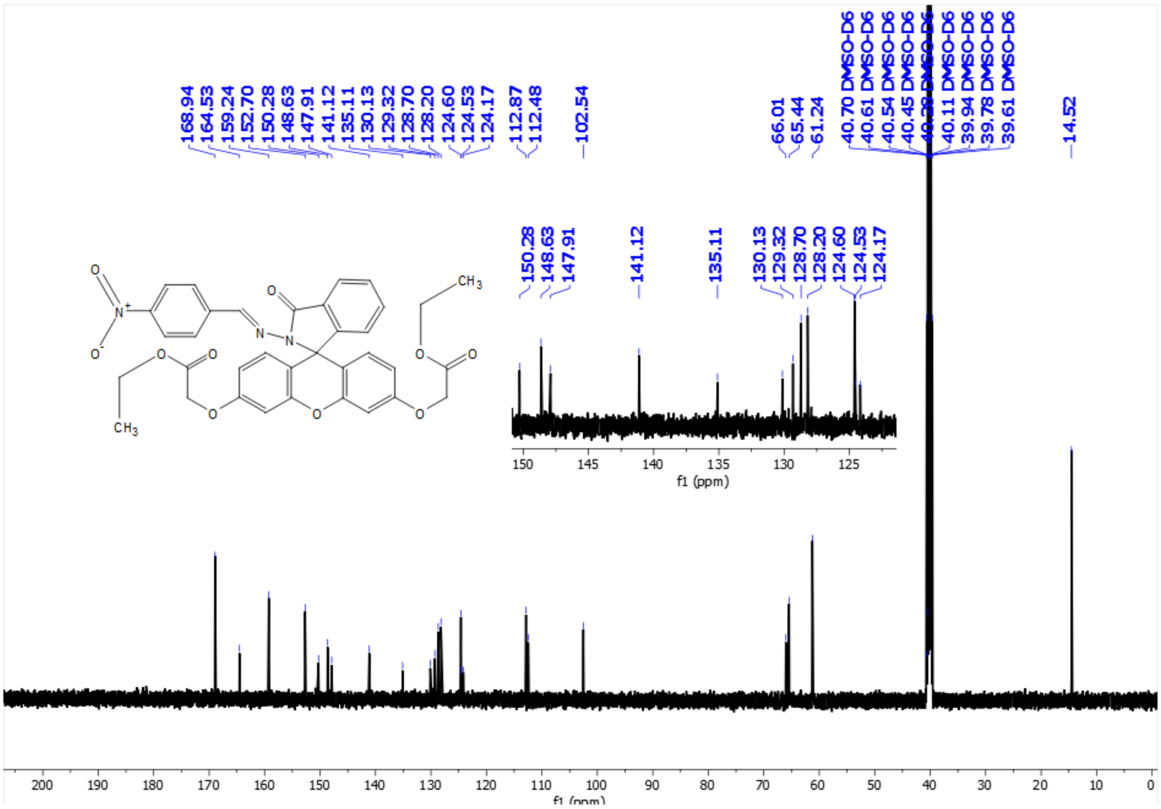


**Figure S 11.** $^{13}$**C NMR spectrum (300 MHz, DMSO-d6) of F2**

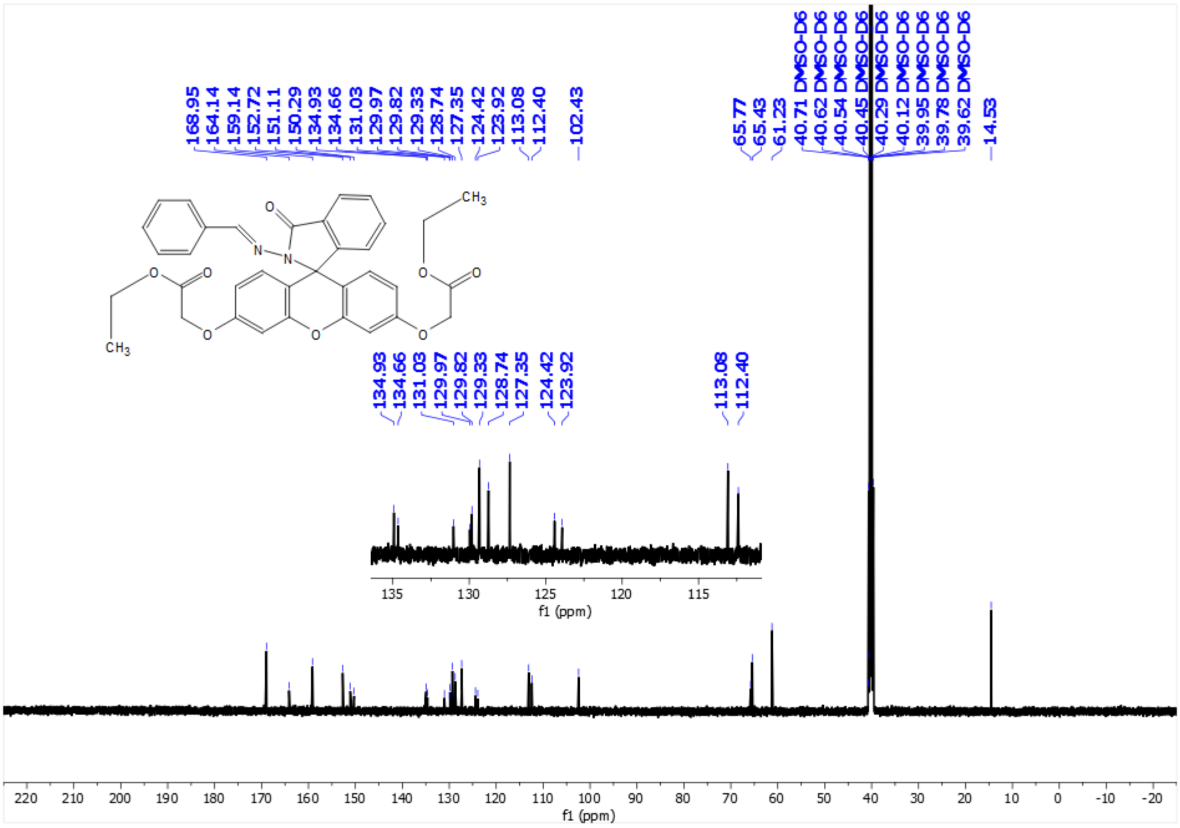


**Figure S 12.** $^{13}$**C NMR spectrum (125 MHz, DMSO-d6) of F3**

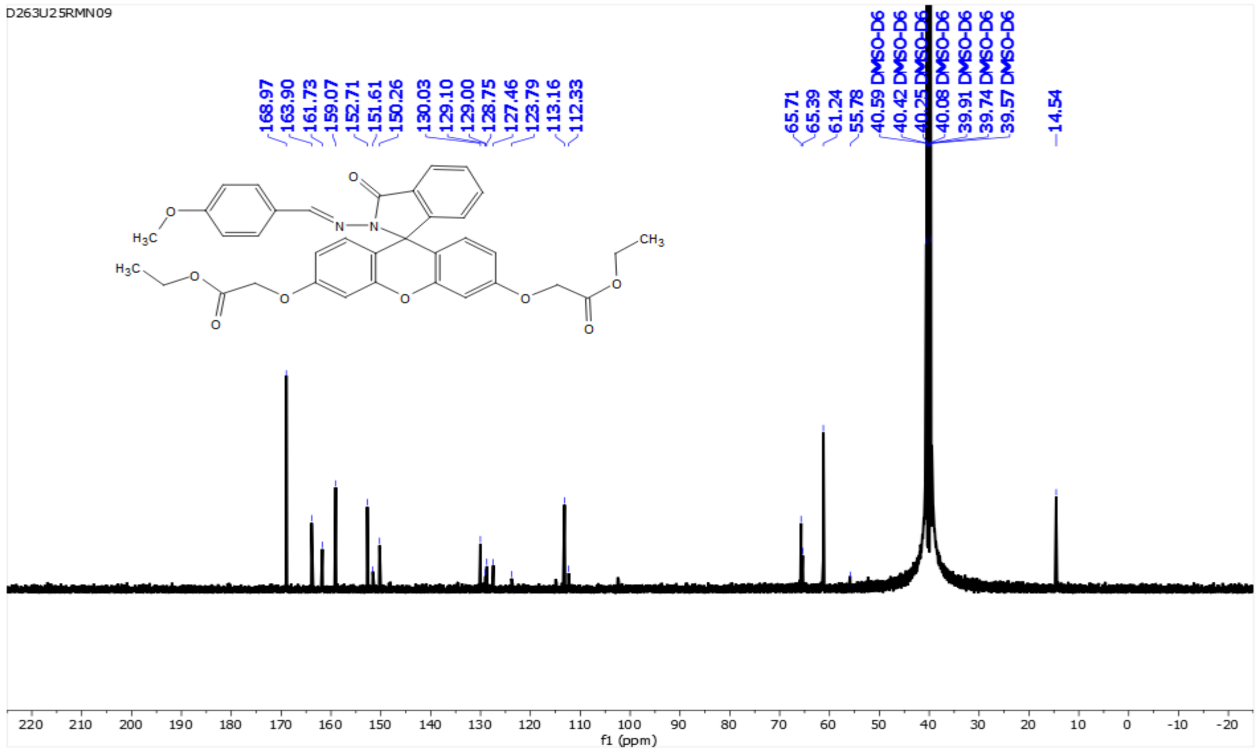


**Figure S 13. $^{13}$C NMR spectrum (125 MHz, DMSO-d6) of F4**

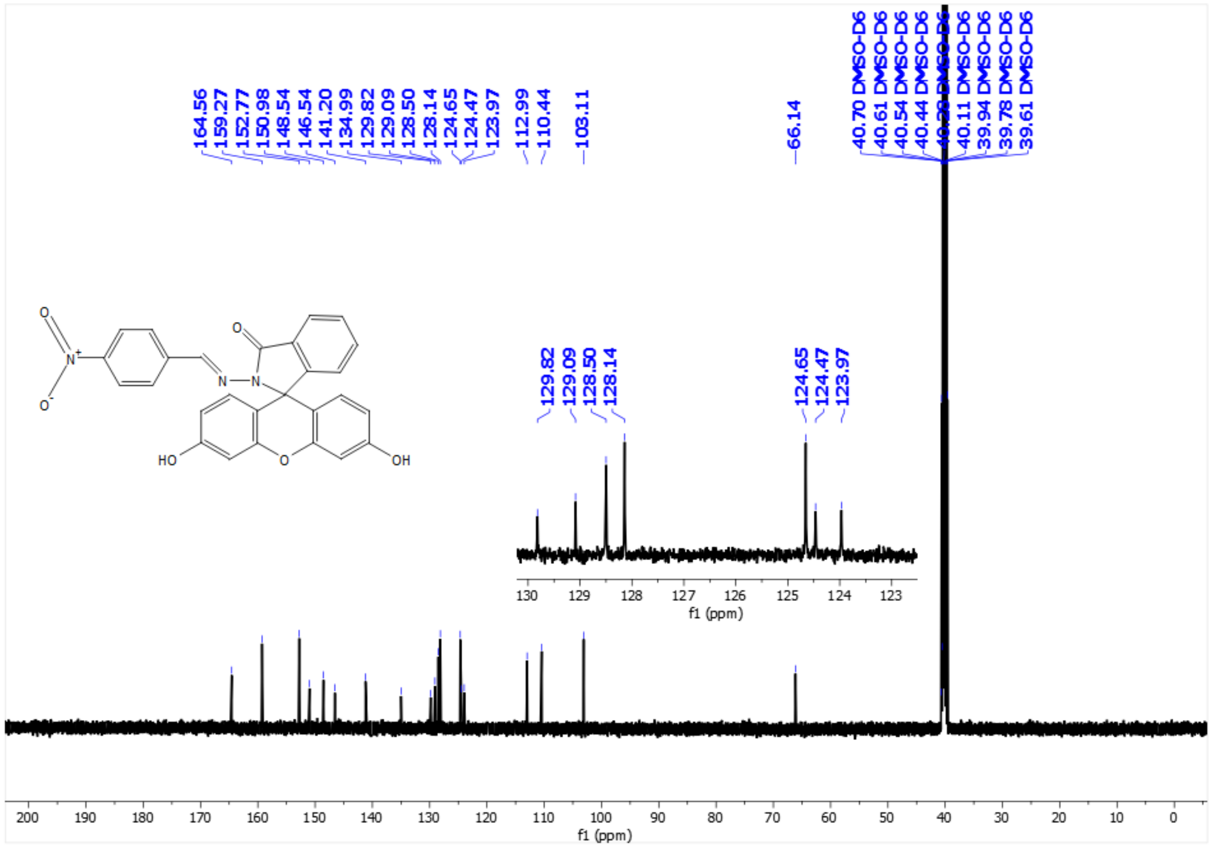


**Figure S 14. $^{13}$C NMR spectrum (125 MHz, DMSO-d6) of F5**

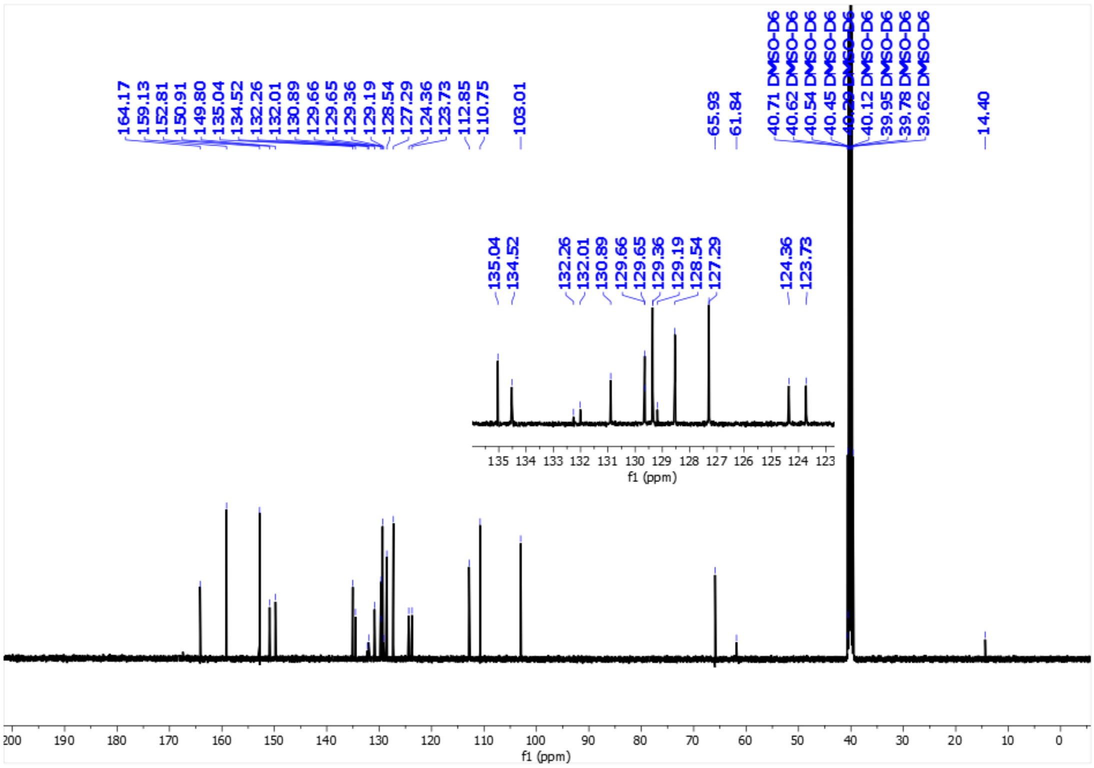


**Figure S 15. $^{13}$C NMR spectrum (125 MHz, DMSO-d6) of F6**

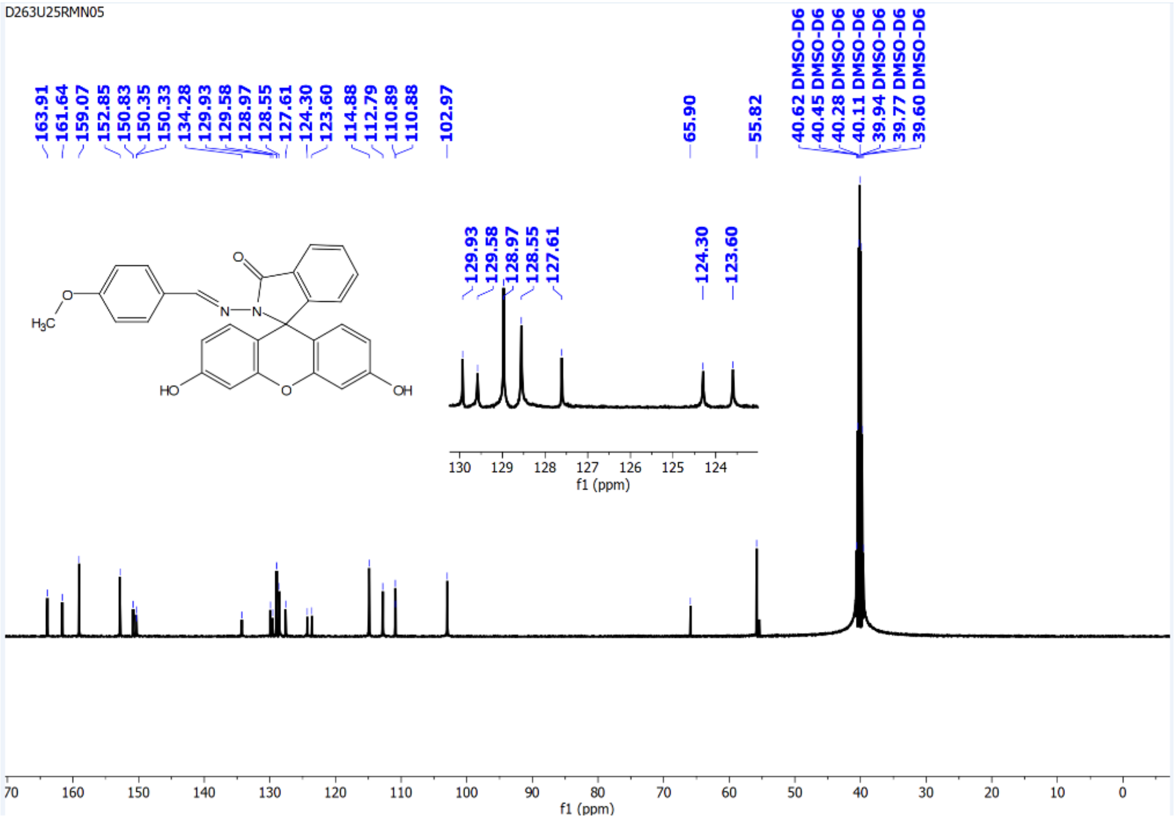


**Figure S 16. $^{13}$C NMR spectrum (125 MHz, DMSO-d6) of F7**

## IV. *HRMS spectra*

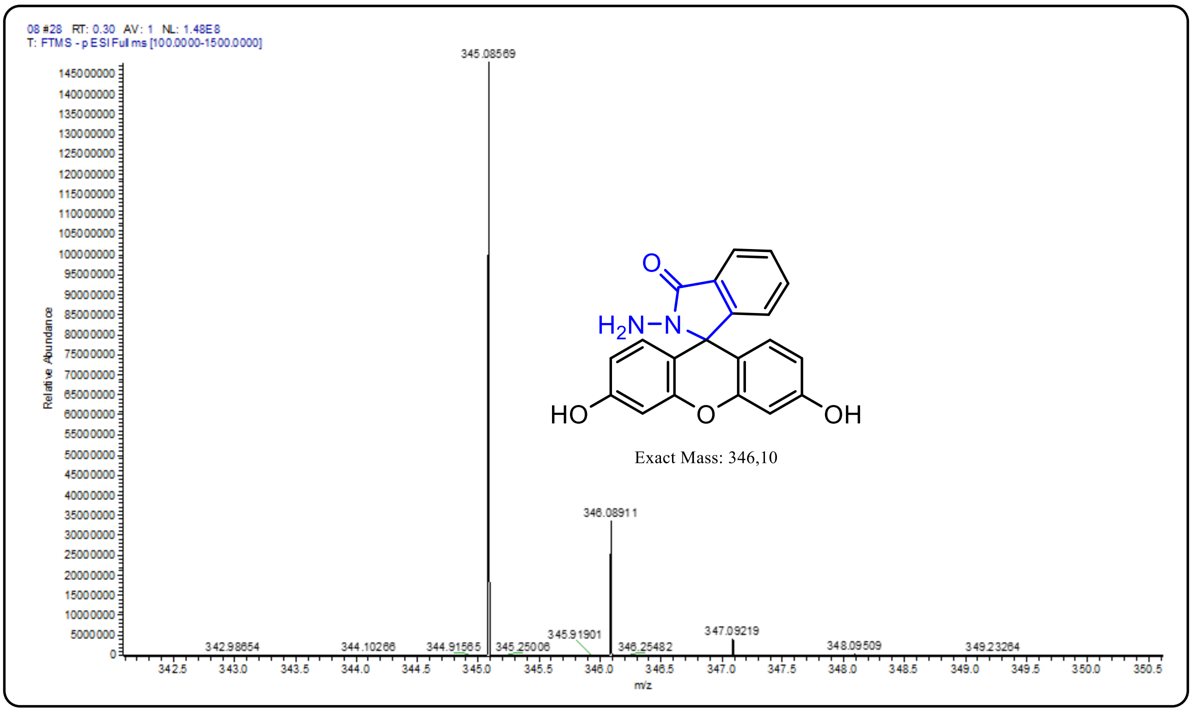


**Figure S 17. Mass spectrum of FH**

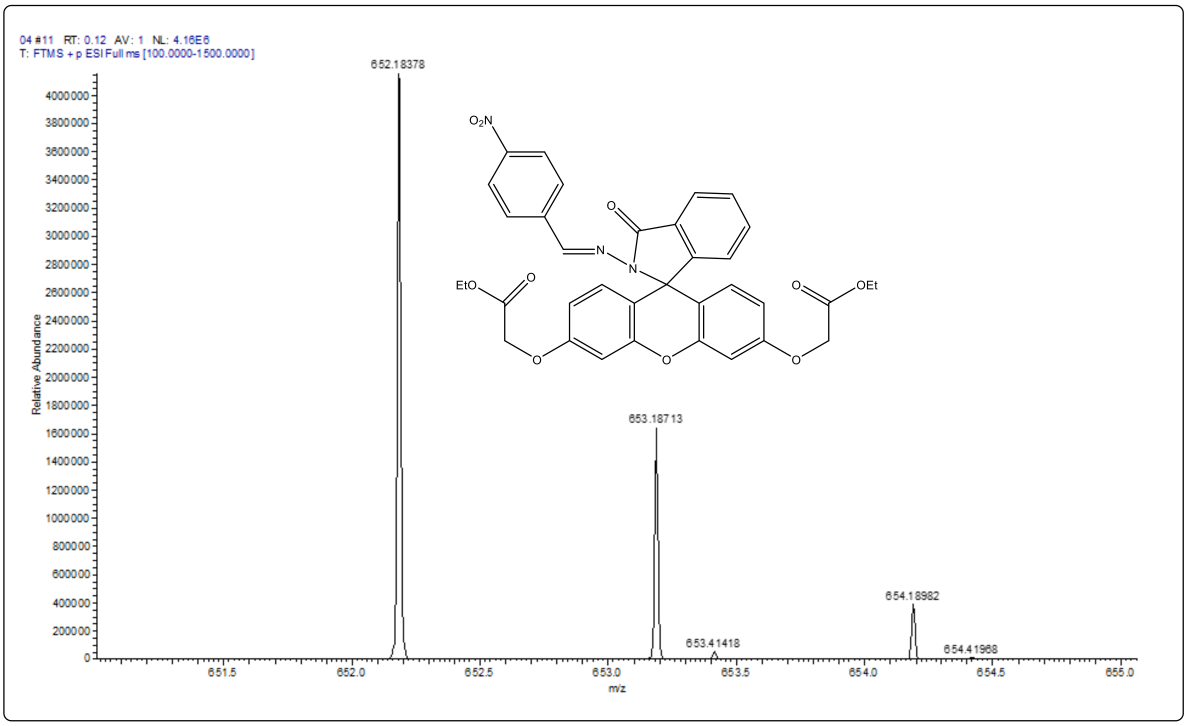


**Figure S 18. Mass Spectrum of F2**

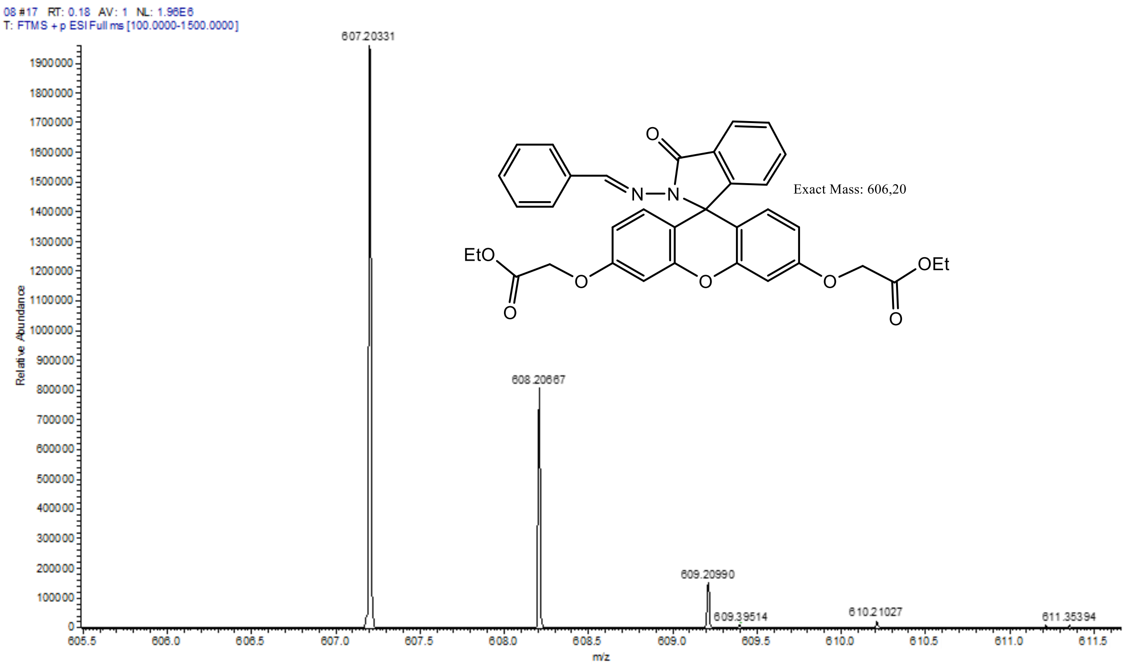


**Figure S 19. Mass Spectrum of F3**

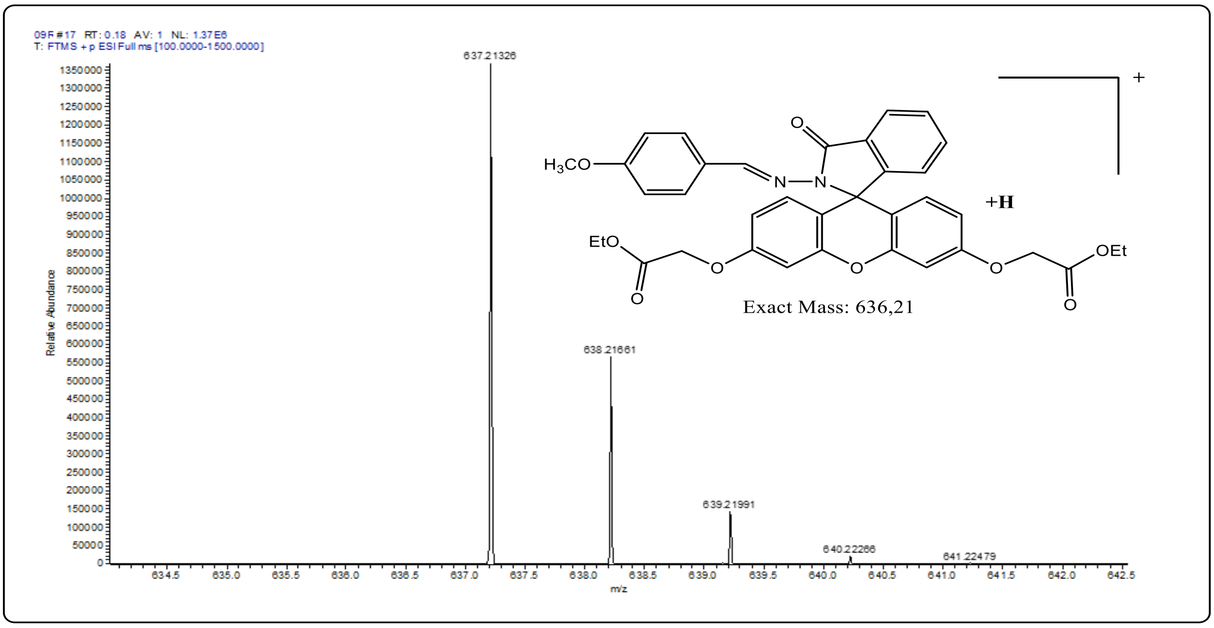


**Figure S 20. Mass Spectrum of F4**

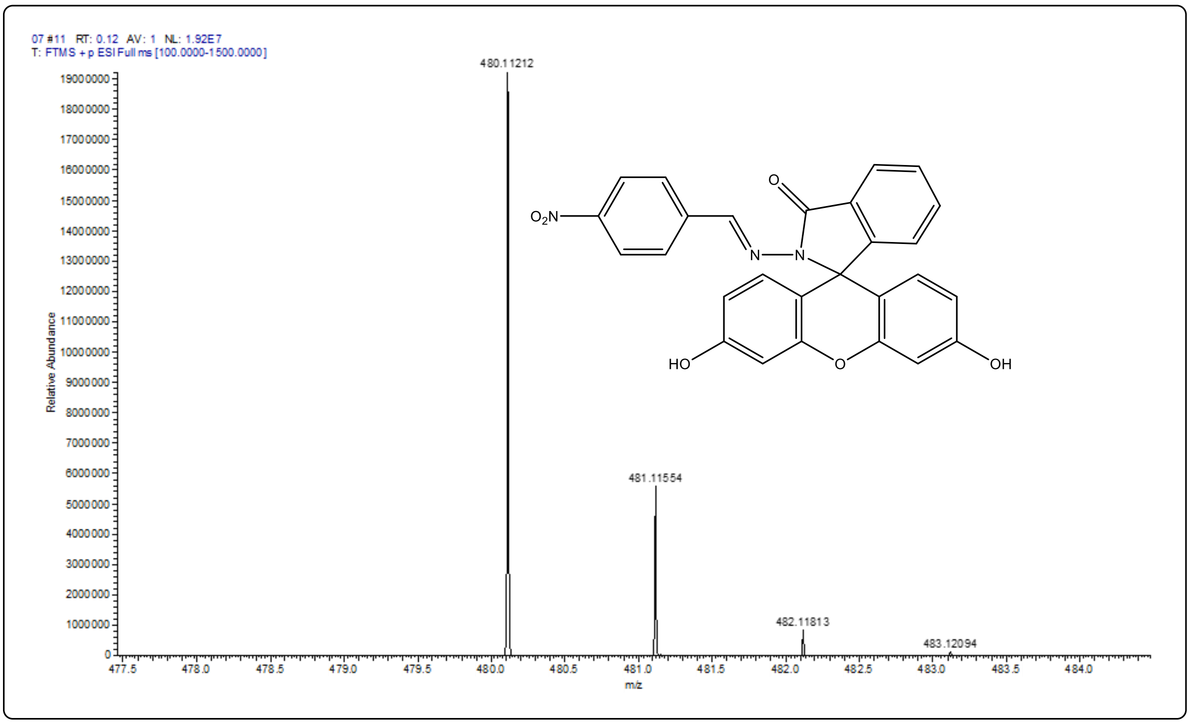


**Figure S 21. Mass Spectrum of F5**

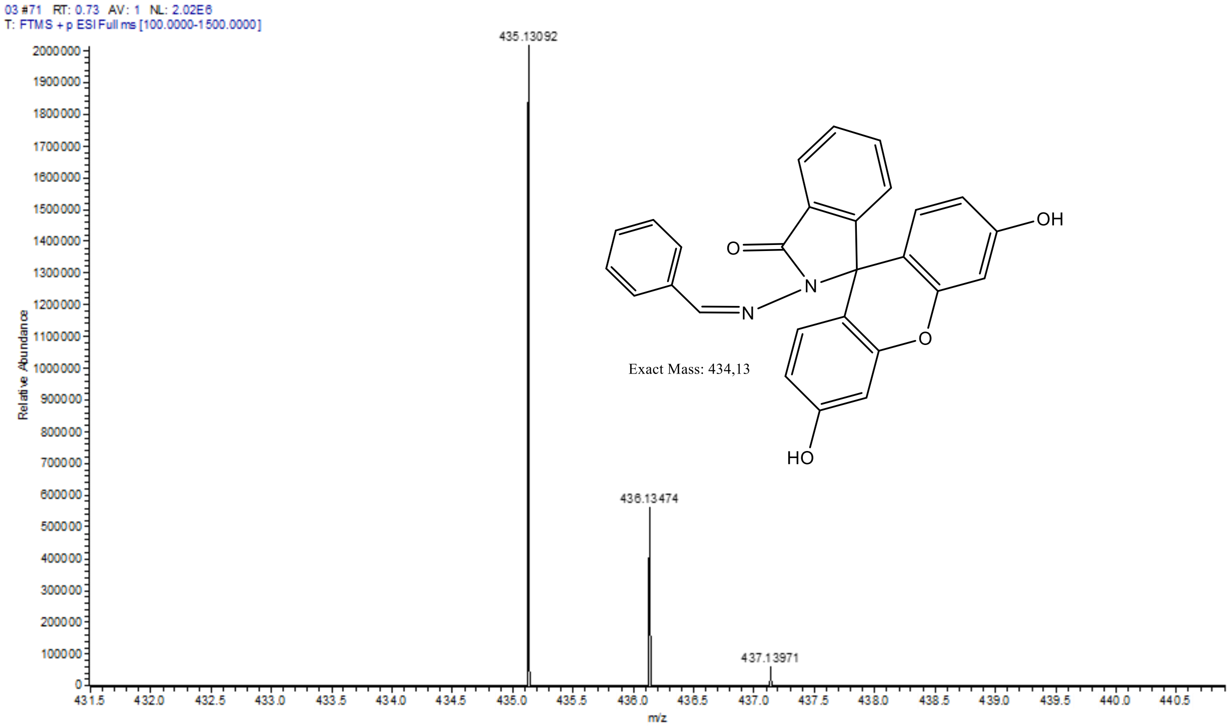


**Figure S 22. Mass Spectrum of F6**

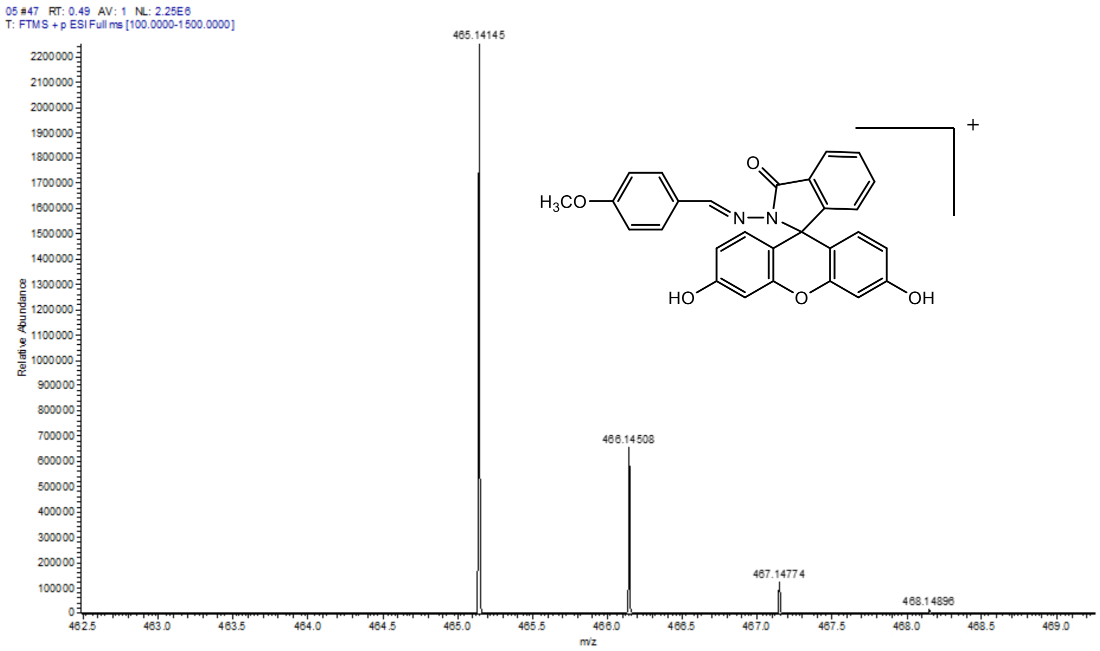


**Figure S 23. Mass Spectrum of F7**
